# Graphene oxide-induced multiple iPSC spheroid formation for enhanced production of angiogenic exosomes accelerating tissue regeneration

**DOI:** 10.1016/j.mtbio.2026.103723

**Published:** 2026-10-05

**Authors:** Woochan Kim, Harshita Sharma, Dream Kim, Shinyull Lee, Sefali Bhakuni, Jangho Kim

**Affiliations:** a Department of Convergence Biosystems Engineering, Chonnam National University, Gwangju, 61186, Republic of Korea; b Department of Rural and Biosystems Engineering, Chonnam National University, Gwangju, 61186, Republic of Korea; c Interdisciplinary Program in IT-Bio Convergence System, Chonnam National University, Gwangju, 61186, Republic of Korea; d Institute of Nano-Stem Cells Therapeutics, NANOBIOSYSTEM Co., Ltd., Gwangju, 61003, Republic of Korea

**Keywords:** Exosomes, Induced pluripotent stem cells, Spheroids, Graphene oxide, Angiogenesis, Tissue regeneration

## Abstract

Exosome-based therapeutics have emerged as a promising cell-free strategy for tissue regeneration by coordinating angiogenesis and tissue repair. Induced pluripotent stem cell (iPSC)-derived exosomes are particularly attractive because of their regenerative potential and scalability; however, the reproducible and scalable production of functionally potent exosomes remains a major barrier to clinical translation. Here, we developed a graphene oxide (GO)-mediated nanoengineering strategy to establish a multi-spheroid platform that simultaneously regulates spheroid architecture, enhances exosome production, and improves angiogenic functionality in both iPSC spheroids and iPSC-derived vascular endothelial cell (vEC) spheroids. GO reproducibly reorganized cellular aggregation from a single dominant spheroid into multiple uniformly sized spheroids per well and increased exosome production while preserving characteristic vesicle properties. GO-engineered iPSC spheroid-derived exosomes exhibited distinct angiogenesis-related miRNA profiles and significantly enhanced endothelial cell migration and capillary-like network formation in vitro. Extending this strategy to iPSC-vEC spheroids further enhanced endothelial differentiation and generated lineage-specific exosomes with greater pro-angiogenic activity. In a rat full-thickness skin wound model, GO-engineered spheroid-derived exosomes accelerated wound closure and promoted vascularized skin regeneration. Collectively, these findings establish GO-mediated spheroid nanoengineering as a reproducible and potentially scalable strategy for producing therapeutically potent exosomes. By integrating controlled spheroid formation, enhanced exosome productivity, preserved vesicle characteristics, and improved angiogenic efficacy, this platform provides a practical framework for standardized exosome manufacturing and supports its future clinical translation toward vascular regenerative therapies.

## Highlights

• Graphene oxide (GO) induces the formation of multiple uniform iPSC spheroids.
• GO-mediated spheroids boost exosome yield while preserving vesicle integrity.
• GO enables iPSC-vEC lineage engineering for potent angiogenic exosome production.
• GO-engineered exosomes enhance the HUVEC angiogenic activity.
• GO-engineered exosomes accelerate vascularized skin tissue regeneration.

## Graphical abstract

**Figure:**
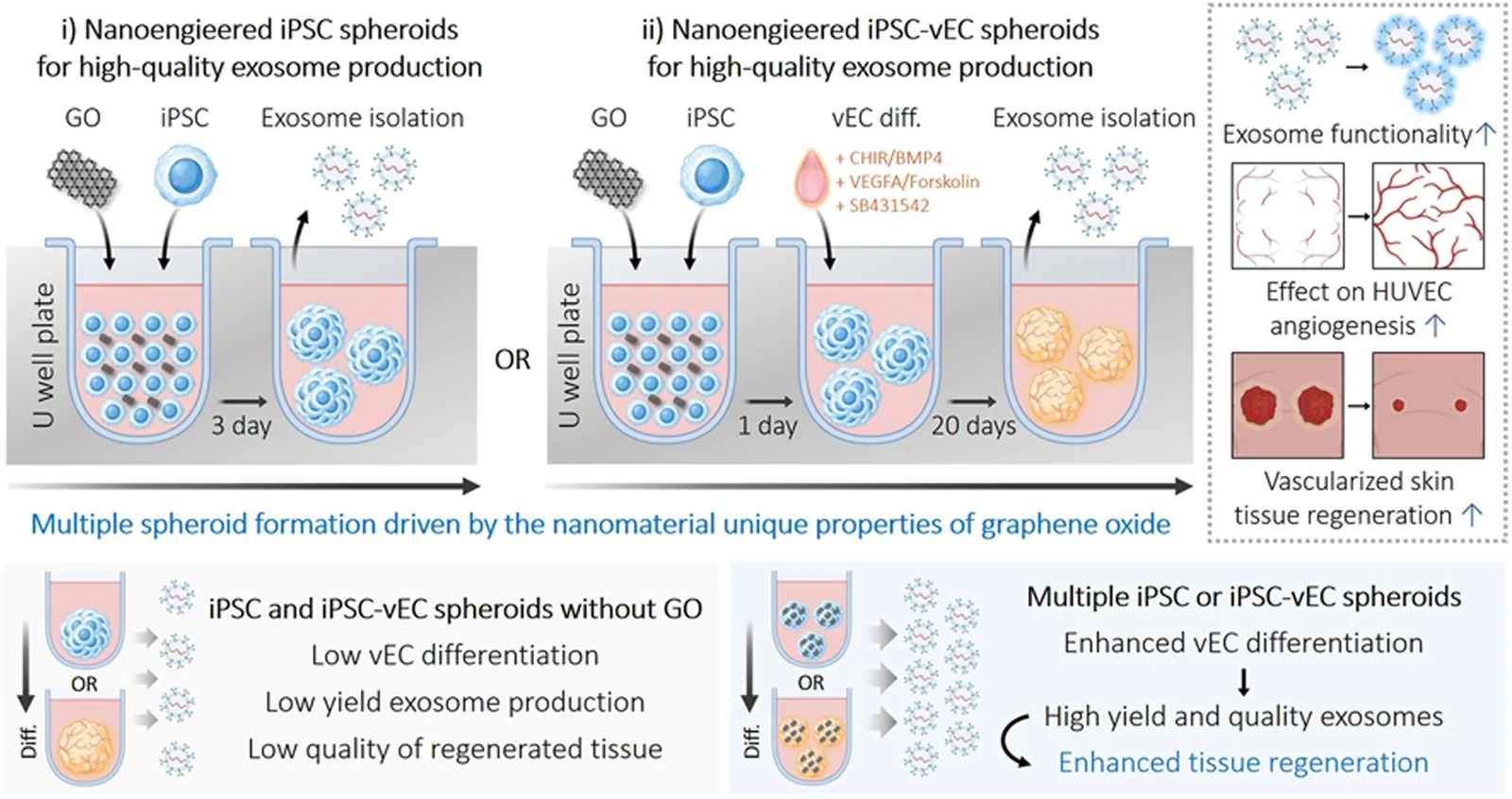

## 1 Introduction

Regenerative medicine is increasingly shifting from conventional stem cell-based therapies toward cell-free therapeutic strategies that retain regenerative efficacy while minimizing safety and translational concerns [[1], [2], [3]]. Although stem cell transplantation has shown substantial promise for tissue repair and functional recovery, its clinical application remains limited by several intrinsic challenges, including low engraftment efficiency, tumorigenic risk, genomic instability, and potential immune rejection [4]. In this context, exosomes, nanosized extracellular vesicles secreted by donor cells, have emerged as a highly promising cell-free therapeutic platform. Exosomes recapitulate many of the regenerative and paracrine benefits of stem cells while offering key practical and safety advantages, including lower immunogenicity, reduced risk of tumor formation or genetic instability, and greater potential for controlled engineering and targeted cargo delivery [[5], [6], [7], [8]]. Owing to their high biocompatibility and potent therapeutic effects, exosomes are increasingly recognized as attractive alternatives for regenerative medicine, particularly in applications requiring coordinated angiogenesis, tissue repair, and extracellular matrix (ECM) remodeling [[9], [10], [11]]. Angiogenesis is essential for tissue regeneration by supplying oxygen, nutrients, and regenerative signals to injured tissues. Impaired vascularization can delay tissue repair, particularly in large or chronic wounds. Therefore, strategies that promote both vascular regeneration and tissue repair are highly relevant to regenerative medicine. Given the importance of angiogenesis in tissue regeneration, successful clinical translation of exosome-based therapies requires both strong pro-angiogenic activity and scalable, reproducible production. However, current manufacturing is limited by low vesicle yield, donor-cell heterogeneity, and inconsistent bioactivity, highlighting the need to improve both exosome productivity and functional potency [12,13].

Conventional exosome manufacturing is predominantly based on two-dimensional (2D) monolayer and suspension culture systems, which remain limited in their ability to support scalable and reproducible therapeutic exosome production [14]. These approaches typically require large culture areas, high media consumption, and extensive processing infrastructure, thereby restricting cost-effectiveness and translational scalability [15]. To address these challenges, a range of bioengineering strategies has been explored to enhance both exosome yield and functional quality, including their pro-angiogenic potency [12,16]. Among these, modulation of the cellular microenvironment through three-dimensional (3D) culture, biochemical stimulation, physical cues, physiologically relevant conditioning, and genetic engineering has emerged as a particularly effective approach [17,18]. Notably, physical and materials-based stimulation strategies offer several translational advantages, including reduced residual toxicity, simplified scale-up, reproducible parameter control, streamlined downstream processing, and improved time- and cost-efficiency [[19], [20], [21]]. In particular, nanoengineered biomaterials provide a versatile and scalable platform to regulate the exosome-producing niche by tailoring biophysical and biochemical cues such as nanotopography, stiffness, viscoelasticity, ligand presentation, and cell-material interactions [22,23]. These microenvironmental cues can directly influence donor-cell mechanotransduction, membrane trafficking, and vesicle biogenesis pathways, thereby modulating both exosome secretion and cargo composition [22,24]. Importantly, such microenvironmental regulation may also alter the enrichment of angiogenesis-related proteins, miRNAs, and signaling molecules within exosomes, thereby enhancing their capacity to promote endothelial migration, vascular network formation, and neovascularization required for effective tissue regeneration. Supporting this concept, Park et al. demonstrated that surface-modified poly(lactic-co-glycolic) acid (PLGA)-incorporated iron oxide nanoparticles enhance mesenchymal stem cell (MSC)-derived exosome release through clathrin-mediated uptake and activation of autophagy/late-endosome (Rab7)-associated trafficking [25]. Similarly, Yang et al. showed that intracellular magnetic iron oxide nanoparticles upregulate gene programs involved in exosome transport, secretion, and biogenesis [26]. In addition, Gurunathan et al. reported that graphene oxide (GO) promotes exosome biogenesis and release in SKOV3 cells [27], while De Souza et al. demonstrated that TiO_2_ nanoparticles stimulate exosome secretion from osteoblasts and alter exosomal cargo composition [28]. Collectively, these studies highlight nanoengineered biomaterials as rational and low-toxicity tools for enhancing exosome productivity and functional programming through regulation of the donor-cell microenvironment.

Among stem cell sources, induced pluripotent stem cells (iPSCs) represent a particularly attractive platform for cell-free exosome production because they combine the regenerative versatility of pluripotent stem cells with the practical advantages of a renewable, expandable, and programmable donor-cell source [12]. To overcome these limitations, three-dimensional (3D) spheroid culture has been increasingly adopted as a strategy to better recapitulate the native cellular microenvironment by enhancing cell-cell interactions, metabolic gradients, and endogenous paracrine signaling [29]. Compared with conventional 2D culture, spheroid-based systems enhance stem cell function, endothelial differentiation, and angiogenic signaling, making them attractive platforms for generating pro-angiogenic exosomes for vascularized tissue regeneration [[30], [31], [32]]. Nevertheless, conventional spheroid systems still often produce a single dominant aggregate per well, which may constrain secretory throughput, structural reproducibility, and overall manufacturing efficiency [33]. Incorporating GO into iPSC culture platforms offers a promising materials-based strategy to regulate spheroid formation and improve the exosome-producing microenvironment [34,35]. Mechanistically, GO provides a high-surface-area interface enriched with oxygen-containing functional groups capable of adsorbing serum proteins and ECM ligands, thereby reinforcing cell-material interactions and stabilizing early multicellular aggregation [36,37]. This ligand-enriched nanoenvironment can strengthen integrin engagement and cytoskeletal organization, promoting more controlled spheroid assembly, improved mechanical integrity, and reduced fragmentation under dynamic culture conditions [35]. In addition, GO can modulate key biophysical parameters, including stiffness, nanoscale roughness, and interfacial energy, thereby influencing mechanotransduction pathways associated with lineage commitment, differentiation, and secretory activity [36]. Despite these advantageous properties, GO has not yet been systematically exploited to engineer multi-spheroid iPSC platforms for the simultaneous enhancement of exosome yield and angiogenic functional potency. To the best of our knowledge, this represents the first GO-based nanoengineering strategy designed to structurally program iPSC spheroid assembly for scalable and functionally enhanced exosome production.

In this study, we developed a GO-mediated nanoengineering strategy to regulate multi-spheroid formation and enhance the production and angiogenic functionality of exosomes derived from iPSC and iPSC-derived vascular endothelial cell (vEC) spheroids. We hypothesized that GO-mediated modulation of spheroid architecture would create a favorable microenvironment for improving endothelial differentiation, exosome biogenesis, and angiogenic bioactivity. We therefore investigated GO-induced changes in spheroid formation, exosome production and characteristics, and angiogenesis-related miRNA profiles, and further extended this strategy to iPSC-vEC spheroids to generate lineage-specific exosomes with enhanced vascular regenerative potential. GO promoted multiple spheroid formation, increased exosome production, and enhanced endothelial differentiation, while GO-engineered exosomes exhibited increased pro-angiogenic activity, including endothelial migration and tube formation. Furthermore, iPSC-vEC spheroid-derived exosomes promoted vascularized skin regeneration in a full-thickness wound model. Collectively, this study establishes a GO-based multi-spheroid platform that simultaneously enhances exosome manufacturing efficiency and angiogenic therapeutic potency for vascularized tissue regeneration.

## 2 Results

### 2.1 GO stimulation promotes efficient iPSC and iPSC-vEC spheroid formation

To establish a structural platform for enhanced exosome generation, we first investigated whether GO incorporation could modulate iPSC aggregation and promote enhanced spheroid formation with greater reproducibility. Prior to biological evaluation, the physicochemical properties of the GO nanosheets were characterized using TEM, zeta potential analysis, FT-IR, XPS, Raman spectroscopy, and AFM (Fig. S1). TEM images showed thin, sheet-like GO nanosheets with lateral dimensions of approximately 300-400 nm, confirming the typical two-dimensional morphology of GO. (Fig. S1A). Zeta potential analysis revealed a negatively charged surface potential of approximately −5.62 mV for GO in aqueous suspension, consistent with the presence of ionizable oxygen-containing functional groups on the GO surface (Fig. S1B). Although this measurement was performed in aqueous suspension rather than complete cell culture medium, it confirms the negative surface charge characteristics of GO; the colloidal behavior of GO may differ under culture conditions because of interactions with salts, proteins, and other medium components (Fig. S1B). FT-IR spectroscopy further confirmed the oxygenated surface chemistry of GO, showing a broad O-H stretching band at approximately 3340 cm^−1^, a C-H stretching band at approximately 2951 cm^−1^, a C=O stretching band at approximately 1732 cm^−1^, a C=C skeletal vibration band at approximately 1620 cm^−1^, a C-OH bending vibration band at approximately 1410 cm^−1^ and C-O/C-O-C-related bands at approximately 1055 cm^−1^. (Fig. S1C). XPS survey analysis confirmed the elemental composition of GO, showing dominant C 1s and O 1s peaks at approximately 285 and 532 eV, respectively (Fig. S1D). High-resolution C 1s spectra were deconvoluted into carbon-related bonding states, including C-C/C=C, C-O, C=O, and O-C=O components, while high-resolution O 1s spectra further confirmed oxygenated carbon species on the GO surface (Fig. S1E and F). Raman spectroscopy displayed the characteristic D and G bands at approximately 1367 and 1598 cm^−1^, respectively. (Fig. S1G). AFM imaging further confirmed the thin nanosheet morphology of GO, with a measured thickness of approximately 1.7 nm based on the corresponding height profile (Fig. S1H and I).

We next evaluated the effects of different GO concentrations on iPSC viability and spheroid formation. iPSCs were cultured with 0, 5, 10, or 20 μg/mL GO under both monolayer and spheroid culture conditions (Fig. S2A). In monolayer culture, cell viability remained high across the tested concentrations at days 1 and 3, with no evident cytotoxicity induced by GO treatment (Fig. S2B and C). Similarly, GO-treated spheroids maintained high viability across the tested concentrations (Fig. S2D and E). Notably, GO incorporation promoted the formation of multiple cellular aggregates, with the effect becoming more apparent at higher GO concentrations. Based on the favorable cellular compatibility and enhanced spheroid-forming behavior, 20 μg/mL GO was selected for subsequent experiments.

Using the selected concentration, we next compared conventional iPSC spheroids with GO-engineered multiple spheroids (GO-M). Conventional culture predominantly generated a single large spheroid per well, whereas GO incorporation resulted in multiple smaller spheroids within the same well (Fig. 1A and C). This GO-mediated aggregation pattern was also applied to iPSC-vEC spheroids, generating GO-MV spheroids for subsequent endothelial differentiation and exosome production (Fig. 1B and D). Bright-field imaging further demonstrated the distinct spheroid organization between the two groups. At day 1, control iPSCs predominantly formed a single large aggregate, whereas GO-M cultures generated multiple smaller spheroids (Fig. 1E and F). Quantitative analysis showed that GO-M spheroids had significantly smaller diameters than conventional iPSC spheroids at days 1, 3, and 5 (Fig. 1G). In contrast, the number of spheroids per 100 wells was substantially increased following GO incorporation and remained higher throughout the culture period, resulting in an increased spheroid yield in the GO-M group (Fig. 1H and I; Fig. S2F–H).

**Fig. 1:**
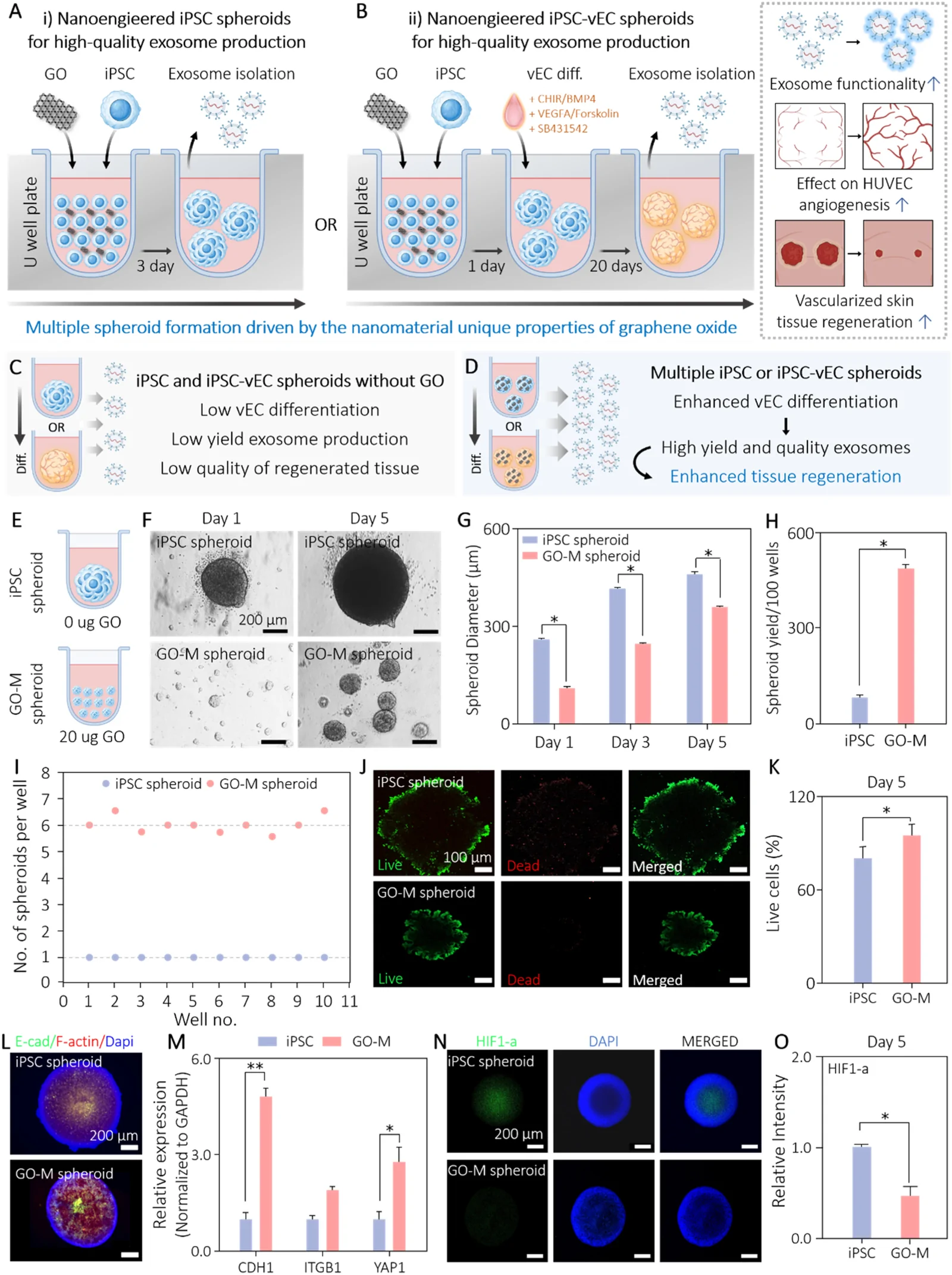
**GO-mediated multiple spheroid formation and characterization of iPSC spheroids. (A)** Schematic illustration of conventional iPSC spheroid formation and GO-mediated multiple spheroid formation (GO-M spheroids). **(B)** Schematic representation of iPSC-vEC spheroid formation with and without GO incorporation and their subsequent use for exosome production and regenerative applications. **(C, D)** Schematic comparison of conventional single-spheroid and GO-mediated multiple-spheroid culture, highlighting the differences in spheroid formation, exosome production, and downstream tissue regeneration. **(E-F)** Schematics and representative bright-field images of conventional iPSC spheroids and GO-M spheroids cultured for 1, 3, and 5 days. Scale bars, 200 μm. **(G)** Quantification of spheroid diameter at days 1, 3, and 5. **(H)** Quantification of the number of spheroids formed per 100 wells on day 5. **(I)** Quantification of the number of spheroids formed per well on day 5. **(J)** Representative fluorescence images showing live/dead staining of conventional iPSC spheroids and GO-M spheroids at day 5. Live cells are shown in green and dead cells in red. Scale bars, 100 μm. **(K)** Quantification of spheroid viability based on live/dead staining. **(L)** Representative F-actin/phalloidin staining of conventional iPSC spheroids and GO-M spheroids at day 5. Scale bars, 200 μm. **(M)** Relative expression of CDH1, ITGB1, and YAP1 in conventional and GO-M spheroids. **(N)** Representative immunofluorescence images of HIF-1α expression in conventional and GO-M spheroids at day 5. Scale bars, 200 μm. **(O)** Quantification of HIF-1α fluorescence intensity. Data are presented as mean ± SD from three independent biological replicates (n = 3), with three technical replicates per condition. (For interpretation of the references to colour in this figure legend, the reader is referred to the Web version of this article.)

We then examined whether the increased spheroid formation was accompanied by changes in cellular viability and organization. Live/dead staining at day 5 showed predominantly viable cells in both control and GO-M spheroids, with no apparent reduction in viability following GO incorporation (Fig. 1J and K). F-actin staining revealed preserved cytoskeletal organization in both groups (Fig. 1L). Analysis of cell adhesion and mechanotransduction-associated markers showed altered expression of *CDH1, ITGB1,* and *YAP1* in GO-M spheroids compared with control spheroids (Fig. 1M). HIF-1α staining also showed a difference between the two groups, with reduced HIF-1α signal observed in GO-M spheroids (Fig. 1N and O). To further examine whether GO-mediated spheroid formation was associated with changes in exosome biogenesis or cellular apoptosis, the expression of exosome-related and apoptosis-associated markers was analyzed. GO-M spheroids exhibited increased expression of *ALIX, SDCBP, TSG101,* and *RAB27A* compared with conventional iPSC spheroids (Fig. S2I). In contrast, the expression of *CASP3, BCL2,* and *BAX* remained comparable between the two groups (Fig. S2J). These observations support the maintenance of cellular homeostasis in GO-M spheroids while suggesting enhanced exosome biogenesis and secretion-related activity.

Collectively, GO incorporation at 20 μg/mL promoted the reproducible formation of multiple smaller iPSC spheroids while maintaining high cellular viability and structural organization. The associated changes in cell adhesion, cytoskeletal, and mechanotransduction-related markers, together with enhanced exosome biogenesis-related activity, support the role of the GO-modulated microenvironment in regulating spheroid architecture and exosome production. These GO-engineered spheroids were subsequently used for further characterization of GO-M-Exo.

### 2.2 GO-engineered iPSC spheroids enhance exosome production and modulate exosomal miRNA cargo

Following the establishment of GO-engineered multiple spheroids, we next investigated whether the increased spheroid formation could enhance exosome production. Conventional iPSC spheroids and GO-engineered multiple spheroids (GO-M spheroids) were cultured under exosome-collection conditions, with the larger number of GO-M spheroids providing an expanded cellular source for exosome secretion (Fig. 2A). Transmission electron microscopy (TEM) revealed vesicular structures with the characteristic morphology of extracellular vesicles in both iPSC spheroid-derived exosomes (IS-Exo) and GO-M spheroid-derived exosomes (GO-M-Exo), with particle sizes in the expected nanoscale range (Fig. 2B). Nanoparticle tracking analysis (NTA) revealed comparable nanoscale particle distributions between IS-Exo and GO-M-Exo (Fig. 2C). When normalized to the initially seeded cell number, GO-M spheroids produced 2.75 × 10^9^ particles/10^6^ cells compared with 1.4 × 10^9^ particles/10^6^ cells for conventional spheroids (Fig. 2D). This increase in particle production was accompanied by a modest difference in the hydrodynamic particle-size distribution, with DLS analysis showing PDI values within a relatively low range for both preparations (Fig. 2E).

**Fig. 2:**
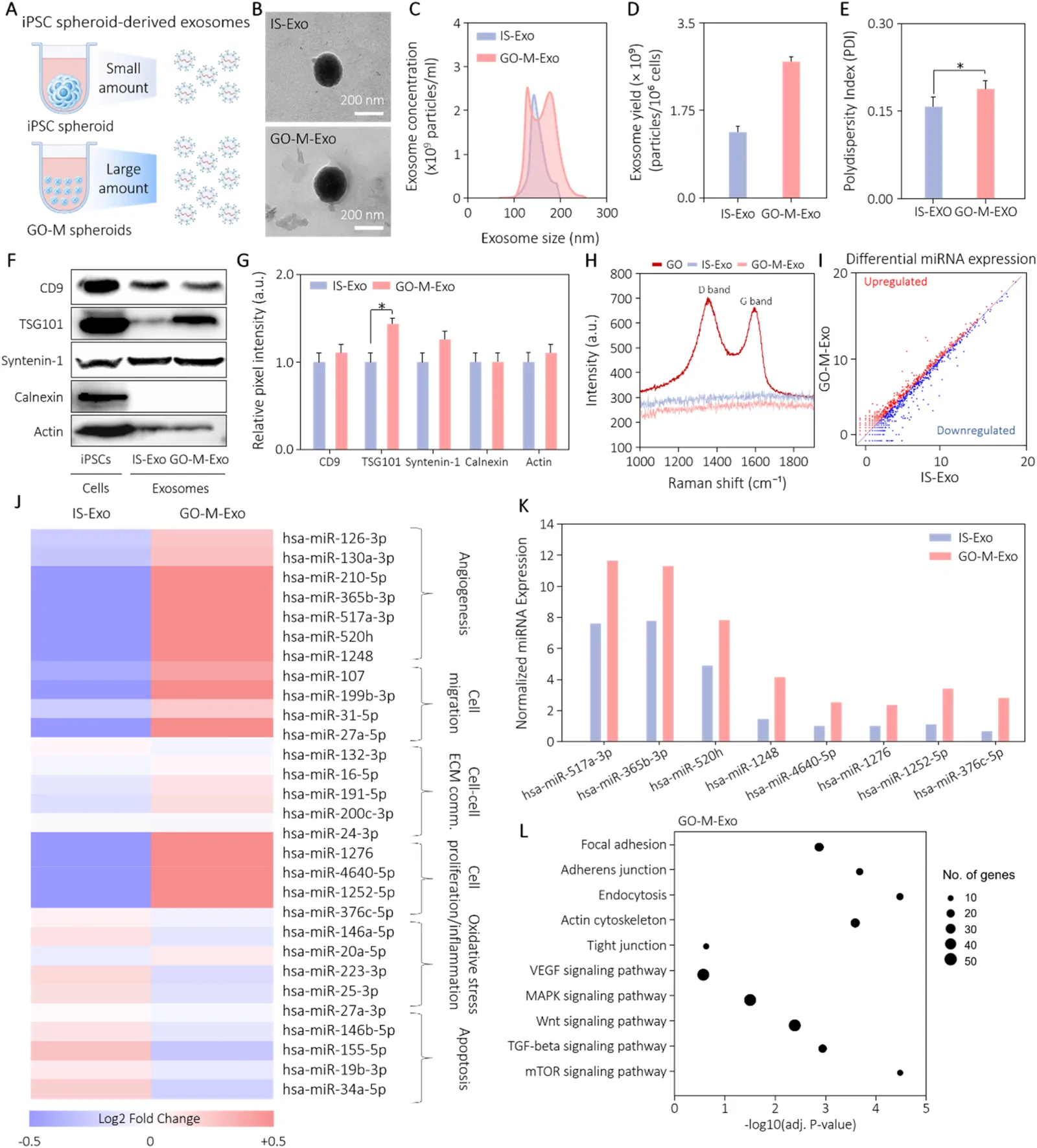
**Characterization and miRNA profiling of exosomes derived from conventional and GO-engineered iPSC spheroids. (A)** Schematic illustration of exosome production from conventional iPSC spheroids and GO-engineered multiple spheroids (GO-M spheroids), highlighting the increased number of exosome-producing spheroids in the GO-engineered condition. **(B)** Representative TEM images of exosomes isolated from conventional iPSC spheroids (IS-Exo) and GO-M spheroids (GO-M-Exo). Scale bars, 200 nm. **(C)** NTA-based particle-size distribution profiles of IS-Exo and GO-M-Exo. **(D)** Quantification of exosome yield normalized to the initially seeded cell number. **(E)** Polydispersity index (PDI) of IS-Exo and GO-M-Exo determined by dynamic light scattering (DLS). **(F)** Representative Western blot analysis of exosomal markers CD9, TSG101, and Syntenin-1, together with Calnexin as a negative marker, in iPSC lysate, IS-Exo, and GO-M-Exo. **(G)** Densitometric quantification of exosomal marker expression. **(H)** Raman spectra of GO, IS-Exo, and GO-M-Exo showing the characteristic D and G bands of GO and the absence of prominent GO-associated Raman features in the isolated exosome preparations. **(I)** Scatter plot showing differential miRNA expression between IS-Exo and GO-M-Exo, with upregulated and downregulated miRNAs indicated. **(J)** Heatmap showing the expression profiles of differentially expressed miRNAs in IS-Exo and GO-M-Exo. **(K)** Relative expression levels of selected differentially expressed miRNAs in IS-Exo and GO-M-Exo. **(L)** KEGG pathway enrichment analysis of experimentally supported target genes of differentially expressed miRNAs. Bubble size represents the number of target genes, and the x-axis represents −log_10_(adjusted p-value). Data are presented as mean ± SD from three independent biological replicates (n = 3), with three technical replicates per condition.

We further examined the molecular characteristics of the isolated vesicles. Western blot analysis detected the canonical exosomal markers CD9, TSG101, and Syntenin-1 in both IS-Exo and GO-M-Exo, whereas the endoplasmic reticulum-associated protein Calnexin was not detected in the exosome preparations (Fig. 2F). Densitometric analysis further confirmed the exosomal marker profiles between the two groups (Fig. 2G). These results support the successful isolation of exosome-enriched vesicular fractions from both conventional and GO-engineered spheroids. To further assess whether GO nanosheets were co-isolated with the exosome preparations, Raman spectroscopy was performed. GO exhibited characteristic D and G bands, whereas neither IS-Exo nor GO-M-Exo displayed the prominent GO-associated Raman features observed in the GO control (Fig. 2H). The absence of an evident GO-specific Raman signature in the isolated vesicle preparations supports the lack of detectable GO nanosheet contamination under the conditions examined.

Since the enhanced exosome production may be accompanied by changes in exosomal cargo, we next investigated the miRNA profiles of IS-Exo and GO-M-Exo. miRNA sequencing revealed distinct expression patterns between the two exosome groups, as demonstrated by the hierarchical clustering of differentially expressed miRNAs (Fig. 2I). Among the differentially expressed miRNAs, several were associated with biological processes relevant to tissue regeneration and angiogenesis. Notably, angiogenesis-associated miRNAs, including hsa-miR-126-3p, hsa-miR-130a-3p, and hsa-miR-210-5p, showed altered expression in GO-M-Exo. In addition, miRNAs associated with cell proliferation, including hsa-miR-199b-3p, hsa-miR-31-5p, and hsa-miR-27a-5p, and those related to cell-ECM communication, including hsa-miR-132-3p and hsa-miR-16-5p, were differentially represented (Fig. 2J). Differential expression analysis further demonstrated a distinct distribution of upregulated and downregulated miRNAs between the two groups (Fig. 2K). To investigate the potential biological functions of these altered miRNA cargos, experimentally supported target genes were subjected to KEGG pathway enrichment analysis. The enriched pathways included focal adhesion, adherens junction, endocytosis, regulation of actin cytoskeleton, tight junction, VEGF signaling, MAPK signaling, Wnt signaling, TGF-β signaling, and mTOR signaling pathways (Fig. 2L). These findings indicate that GO engineering not only enhances exosome production but also modulates exosomal miRNA cargo associated with key pathways involved in cell adhesion, cytoskeletal regulation, and angiogenic signaling, providing a potential molecular basis for the enhanced regenerative activity of GO-M-Exo.

### 2.3 GO-M-Exo enhances endothelial migration and angiogenic activity through VEGFR2-dependent signaling

To determine whether the increased exosome production and altered miRNA cargo of GO-engineered spheroids translated into enhanced endothelial activity, we first evaluated the effects of IS-Exo and GO-M-Exo on HUVEC migration. Exosome treatment was associated with increased expression of angiogenesis-related factors, including ANG-1, ANG-2, HGF, bFGF, and VEGF, with GO-M-Exo showing a more pronounced angiogenic factor profile than IS-Exo (Fig. 3A) [38]. A scratch wound-healing assay was subsequently performed to evaluate endothelial cell migration. Compared with untreated control cells, both IS-Exo and GO-M-Exo promoted wound closure over 24 h, with GO-M-Exo producing the greatest increase in wound coverage (Fig. 3B and C). These results indicate that GO engineering of the parent spheroids enhanced the pro-migratory activity of their secreted exosomes. We next investigated whether GO-M-Exo could enhance endothelial network formation. HUVECs treated with IS-Exo or GO-M-Exo formed more extensive interconnected networks than untreated controls (Fig. 3D). Quantitative analysis demonstrated that GO-M-Exo significantly increased total tube length, total branching length, number of nodes, and number of loops compared with the control and/or IS-Exo groups (Fig. 3E–H).

**Fig. 3:**
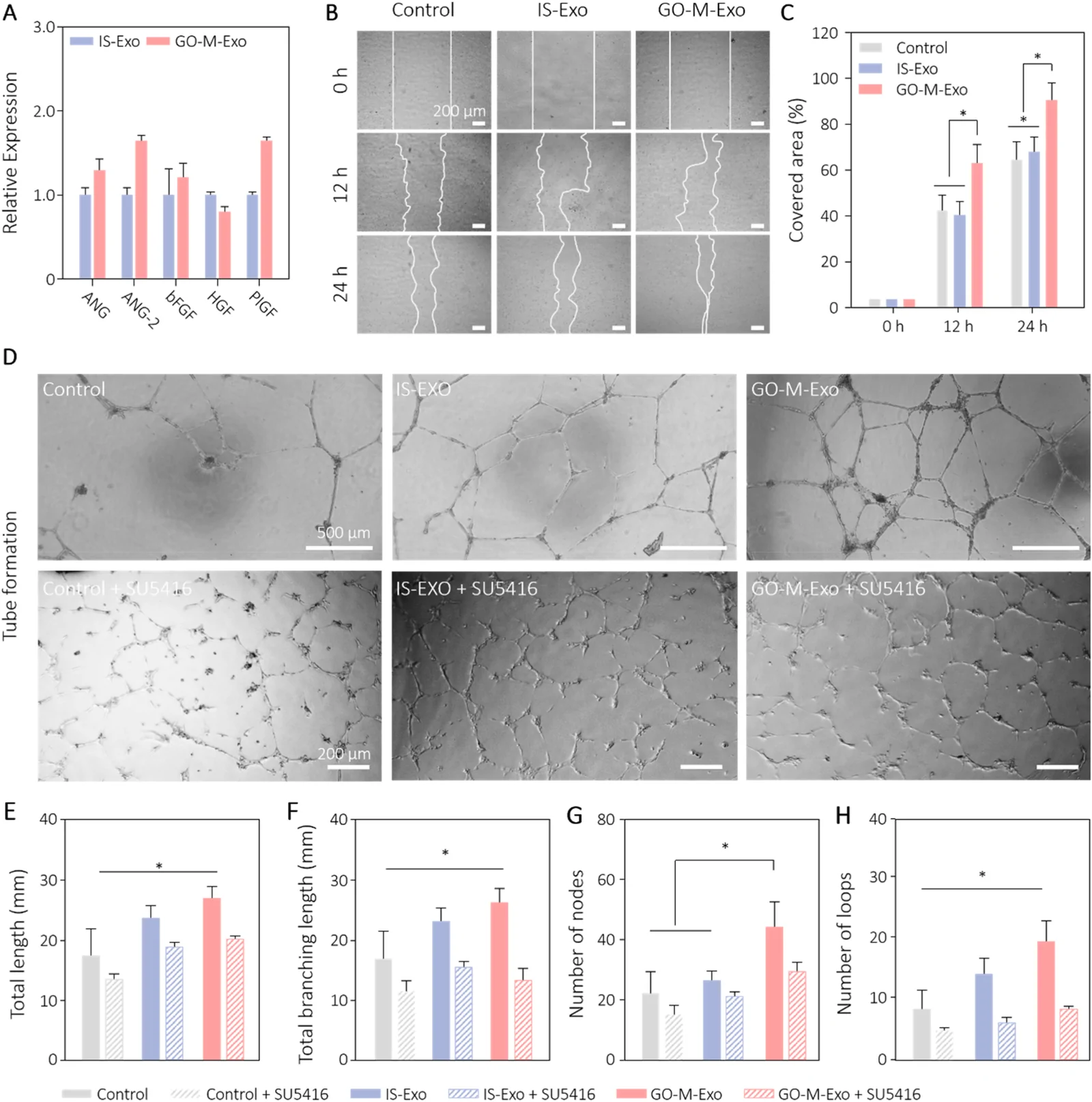
**GO-M-Exo enhances HUVEC migration and angiogenic network formation through VEGFR2-dependent signaling. (A)** Relative expression of angiogenesis-associated factors, including ANG-1, ANG-2, HGF, bFGF, and VEGF, in IS-Exo and GO-M-Exo. **(B)** Representative images of HUVEC scratch wound-healing assays at 0, 12, and 24 h following treatment with Control, IS-Exo, or GO-M-Exo. Scale bars, 200 μm. **(C)** Quantification of wound coverage area at 0, 12, and 24 h. **(D)** Representative images of HUVEC tube formation following treatment with Control, IS-Exo, or GO-M-Exo in the presence or absence of the VEGFR2 inhibitor SU5416. Scale bars, 500 μm for the upper panels and 200 μm for the lower panels. **(E-H)** Quantification of total tube length, total branching length, number of nodes, and number of loops, respectively. SU5416, semaxanib, a pharmacological inhibitor of VEGFR2 signaling. Data are presented as mean ± SD from three independent biological replicates (n = 3), with three technical replicates per condition.

To determine whether VEGFR2 signaling contributed to the enhanced angiogenic activity of GO-M-Exo, the VEGFR2 inhibitor SU5416 was incorporated into the tube-formation assay. While GO-M-Exo markedly enhanced endothelial network formation, inhibition of VEGFR2 signaling attenuated these effects, as evidenced by reduced network formation and associated quantitative parameters in the GO-M-Exo + SU5416 group compared with GO-M-Exo alone (Fig. 3D–H). The attenuation of the GO-M-Exo-mediated angiogenic phenotype by VEGFR2 inhibition supports the involvement of VEGFR2-dependent signaling in the enhanced endothelial response to GO-M-Exo. Collectively, GO-M-Exo exhibited enhanced pro-migratory and pro-angiogenic activity compared with IS-Exo, while pharmacological inhibition of VEGFR2 attenuated the GO-M-Exo-mediated angiogenic response, supporting a VEGFR2-dependent mechanism.

### 2.4 GO-engineered iPSC spheroid-derived exosomes accelerate wound healing in vivo

To determine whether the enhanced angiogenic activity of GO-M-Exo translates into functional tissue repair, a rat full-thickness skin defect model was established (Fig. 4A). Excisional wounds were surgically created on the dorsal skin of rats, followed by local administration of IS-Exo or GO-M-Exo (50 μg per treatment) at days 0, 3, 7, and 10. Wound healing was monitored until day 14.

**Fig. 4:**
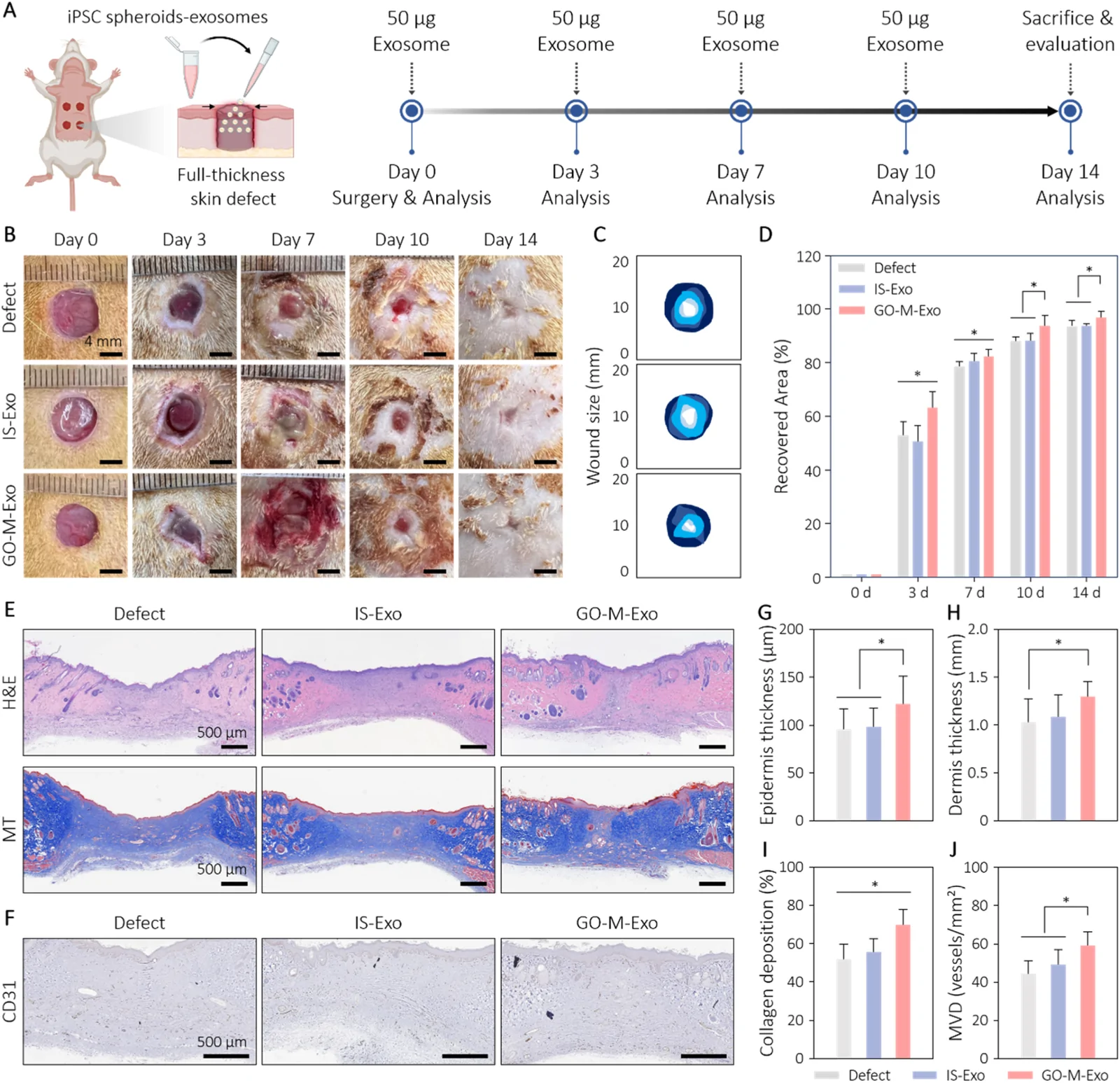
**Therapeutic evaluation of GO-mediated iPSC spheroid-derived exosomes in a rat wound healing model. (A)** Schematic illustration of the in vivo experimental workflow. Full-thickness excisional wounds were generated on the dorsal skin of rats, followed by local administration of IS-Exo or GO-M-Exo (n = 6 animals per group). **(B)** Representative macroscopic images showing wound closure at different time points following treatment with phosphate-buffered saline (PBS; control), IS-Exo, or GO-M-Exo. **(C-D)** Quantitative analysis of wound closure showing wound recovery rates over time. Both exosome-treated groups exhibited accelerated wound healing compared with controls, with GO-M-Exo producing the most pronounced improvement. **(E)** Representative hematoxylin and eosin (H&E)- and Masson's trichrome-stained sections of regenerated wound tissues at day 14. **(F)** Representative CD31 immunohistochemical staining showing CD31-positive vascular structures (brown) within the regenerated wound tissue. **(G)** Quantitative analysis of granulation tissue thickness. **(H)** Quantitative analysis of epidermal thickness. **(I)** Quantification of collagen deposition, expressed as the percentage of collagen-positive area relative to the total analyzed wound tissue area. **(J)** Quantification of CD31-positive MVD, expressed as the number of CD31-positive vessels per unit tissue area (vessels/mm^2^). Quantitative analyses were performed using n = 6 animals per group. (For interpretation of the references to colour in this figure legend, the reader is referred to the Web version of this article.)

Representative macroscopic images revealed clear differences in wound closure dynamics among the experimental groups (Fig. 4B). Untreated wounds exhibited delayed closure with irregular margins and incomplete epithelial coverage, whereas GO-M-Exo-treated wounds showed markedly accelerated healing, with visible wound contraction by day 3 and progressive restoration of the skin surface. By day 14, GO-M-Exo-treated wounds displayed near-complete closure, while untreated wounds remained partially open. Quantitative analysis confirmed significantly reduced residual wound area in the GO-M-Exo group compared with both untreated controls and IS-Exo-treated wounds (Fig. 4C and D). The percentage of wound closure reached approximately 97% in the GO-M-Exo group at day 14, compared with ∼91% in the untreated controls, demonstrating significantly accelerated wound repair. The improved healing observed with GO-M-Exo is consistent with its enhanced angiogenic activity in vitro. Angiogenesis plays a central role in skin repair by supplying oxygen and nutrients and by supporting fibroblast activation, keratinocyte migration, and extracellular matrix remodeling [39]. Given the robust pro-angiogenic factor expression and endothelial activation induced by GO-M-Exo in vitro, the accelerated wound healing observed in vivo is likely associated with enhanced neovascularization during the proliferative phase of tissue repair.

Histological analysis at day 14 further supported the enhanced regenerative effects of GO-M-Exo. H&E staining showed more advanced tissue reconstruction in the GO-M-Exo-treated group than in the untreated control and IS-Exo groups, characterized by increased granulation tissue formation and more complete restoration of the epidermal layer (Fig. 4E). Masson's trichrome staining further revealed greater collagen deposition within the regenerated wound tissue following exosome treatment, with the most pronounced collagen accumulation observed in the GO-M-Exo group (Fig. 4E). In parallel, CD31 immunohistochemical staining showed a higher abundance of CD31-positive vascular structures in GO-M-Exo-treated wounds, suggesting enhanced neovascularization within the regenerating tissue (Fig. 4F). Notably, repeated local administration of IS-Exo and GO-M-Exo did not induce obvious inflammatory cell infiltration, tissue necrosis, or abnormal histological reactions in the regenerated wound tissue at day 14. Consistent with these histological findings, quantitative histomorphometric analysis demonstrated increased granulation tissue thickness and epidermal thickness after exosome treatment, with the GO-M-Exo group exhibiting the highest values among the experimental groups (Fig. 4G and H). These results indicate that GO-M-Exo effectively promoted both dermal tissue regeneration and re-epithelialization during wound repair. Quantification of collagen deposition also showed a higher collagen-positive area in the GO-M-Exo-treated wounds than in the untreated control and IS-Exo groups (Fig. 4I), further indicating enhanced extracellular matrix deposition and tissue remodeling. Likewise, microvessel density (MVD) was increased following exosome treatment and reached its highest level in the GO-M-Exo group (Fig. 4J), corroborating the CD31 staining results and further supporting the pro-angiogenic effect of GO-M-Exo during wound regeneration. Collectively, these histological and immunohistochemical findings demonstrate that GO-M-Exo not only accelerates macroscopic wound closure but also enhances re-epithelialization, granulation tissue formation, collagen deposition, and neovascularization, thereby promoting more effective structural and vascular regeneration of the wounded skin.

### 2.5 GO promotes efficient iPSC-vEC differentiation and endothelial maturation

Following the establishment of GO-engineered iPSC spheroids and their enhanced exosome production, we next investigated whether the GO-mediated multiple-spheroid platform could be extended to endothelial differentiation. iPSC spheroids were subjected to a stepwise differentiation protocol consisting of pluripotency, mesodermal induction, endothelial specification, and maturation stages (Fig. 5A). Conventional iPSC-vEC spheroids (ISV) and GO-engineered multiple iPSC-vEC spheroids (GO-MV) were generated using GO-free and 20 μg/mL GO conditions, respectively (Fig. 5B). Bright-field imaging revealed distinct spheroid organization throughout endothelial differentiation. At day 1, both groups formed cellular aggregates, whereas GO-treated cultures maintained multiple smaller aggregates throughout differentiation and maturation to day 20 (Fig. 5C). At the endpoint, the GO-MV group displayed multiple spheroids within the culture well compared with the predominantly single spheroid observed in the ISV condition (Fig. 5D). Quantitative analysis showed that GO-MV spheroids maintained a smaller spheroid diameter at days 1, 10, and 20 compared with ISV spheroids (Fig. 5E). Consistent with the altered aggregation pattern, the spheroid yield was substantially higher in the GO-MV group (Fig. 5F).

**Fig. 5:**
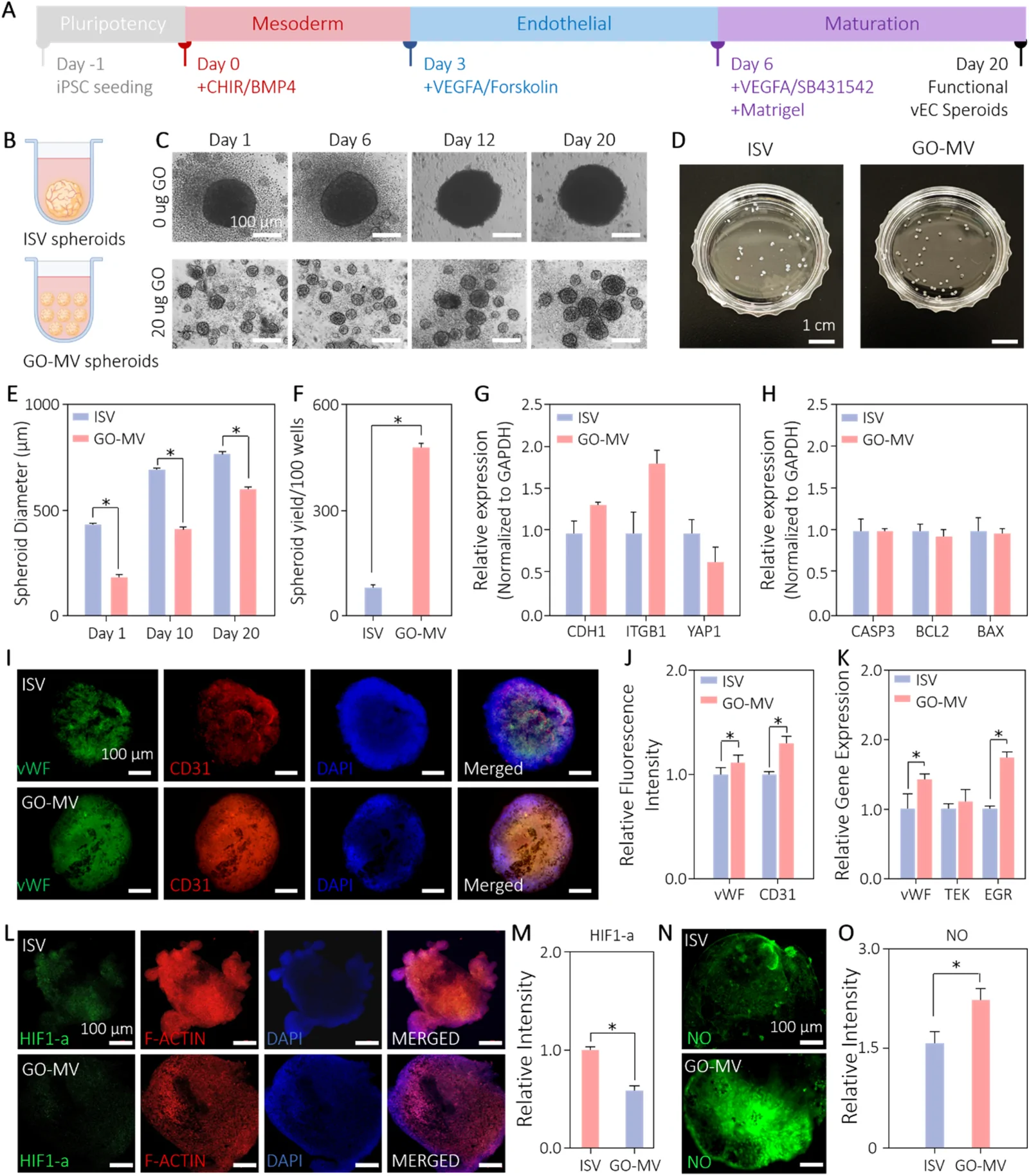
**GO-engineered multiple iPSC-vEC spheroids with enhanced endothelial differentiation and maturation. (A)** Schematic illustration of the stepwise differentiation protocol used to generate iPSC-vEC spheroids, including pluripotency, mesodermal induction, endothelial specification, and maturation stages. **(B)** Schematic representation of conventional iPSC-vEC spheroids (ISV) and GO-engineered multiple iPSC-vEC spheroids (GO-MV). **(C)** Representative bright-field images of ISV and GO-MV spheroids at days 1, 6, 12, and 20 of endothelial differentiation. Scale bars, 100 μm. **(D)** Representative images of ISV and GO-MV spheroid cultures at day 20. Scale bars, 2 mm. **(E)** Quantification of spheroid diameter at days 1, 10, and 20. **(F)** Quantification of spheroid yield per 100 wells at day 20. **(G)** Relative expression of CDH1, ITGB1, and YAP1 in ISV and GO-MV spheroids. **(H)** Relative expression of apoptosis-associated markers CASP3, BCL2, and BAX in ISV and GO-MV spheroids. **(I)** Representative immunofluorescence images of VWF and CD31 expression in ISV and GO-MV spheroids at day 20. Scale bars, 100 μm. **(J)** Quantification of VWF and CD31 fluorescence intensity. **(K)** Relative expression of the endothelial markers VWF, TEK, and EGR determined by qRT-PCR. **(L)** Representative immunofluorescence images showing HIF-1α and F-actin staining in ISV and GO-MV spheroids at day 20. Scale bars, 100 μm. **(M)** Quantification of HIF-1α fluorescence intensity. **(N)** Representative immunofluorescence images of NO expression in ISV and GO-MV spheroids at day 20. **(O)** Quantification of NO fluorescence intensity. Data are presented as mean ± SD from three independent biological replicates (n = 3), with three technical replicates per condition.

We next examined whether GO-mediated spheroid organization affected the expression of cell adhesion and mechanotransduction-associated markers during endothelial differentiation. The expression of *CDH1, ITGB1,* and *YAP1* differed between ISV and GO-MV spheroids, indicating changes in cell-cell and cell-matrix interactions associated with GO-mediated spheroid organization (Fig. 5G). In contrast, the expression of apoptosis-associated markers *CASP3, BCL2,* and *BAX* remained comparable between the two groups (Fig. 5H). To confirm endothelial differentiation and maturation, immunofluorescence staining for the endothelial markers VWF and CD31 was performed at day 20. Both ISV and GO-MV spheroids exhibited positive staining for VWF and CD31, while quantitative analysis and PCR analysis revealed increased endothelial marker expression in GO-MV spheroids (Fig. 5I and J). F-actin staining further demonstrated preserved cellular organization within both spheroid populations (Fig. 5K). HIF-1α staining was also evaluated to assess the hypoxia-associated microenvironment. GO-MV spheroids showed decreased HIF-1α fluorescence compared with ISV spheroids (Fig. 5L and M). To further assess the functional endothelial characteristics of the spheroids, intracellular nitric oxide (NO) production was evaluated using DAF-FM Diacetate. GO-MV spheroids exhibited significantly higher DAF-FM fluorescence intensity compared with conventional ISV spheroids, indicating an enhanced intracellular NO level following GO-mediated spheroid engineering (Fig. 5N and O). This increase in NO production further supports the enhanced endothelial functional phenotype of GO-MV spheroids. Collectively, these results demonstrate that GO incorporation can be extended to iPSC-vEC spheroid generation, promoting the formation of multiple smaller spheroids with increased culture yield while maintaining cellular viability and endothelial differentiation. The enhanced expression of endothelial markers in GO-MV spheroids further supports the use of GO-engineered multiple spheroids as an efficient platform for generating endothelialized spheroids and their derived exosomes.

### 2.6 GO-engineered iPSC-vEC spheroids enhance exosome production with exosomal miRNA cargo modulation

Following the establishment of GO-engineered iPSC-vEC spheroids, we next investigated whether the increased spheroid yield could be translated into enhanced production of vascular endothelial cell-derived exosomes. Exosomes were isolated from conventional iPSC-vEC spheroids (ISV-Exo) and GO-engineered iPSC-vEC spheroids (GO-MV-Exo) under comparable collection conditions (Fig. 6A). TEM analysis revealed nanoscale vesicular structures with the characteristic morphology of extracellular vesicles in both ISV-Exo and GO-MV-Exo preparations (Fig. 6B). NTA showed that both exosome preparations exhibited particle-size distributions within the nanoscale range, with comparable overall size profiles between ISV-Exo and GO-MV-Exo (Fig. 6C). Quantification of particle production demonstrated an increased exosome yield from GO-MV spheroids compared with conventional ISV spheroids (ISV-Exo: 1.6 × 10^9^ particles/10^6^ cells; GO-MV-Exo: 2.82 × 10^9^ particles/10^6^ cells) (Fig. 6D). DLS analysis further showed the hydrodynamic particle-size distribution and PDI of the two preparations (Fig. 6E).

**Fig. 6:**
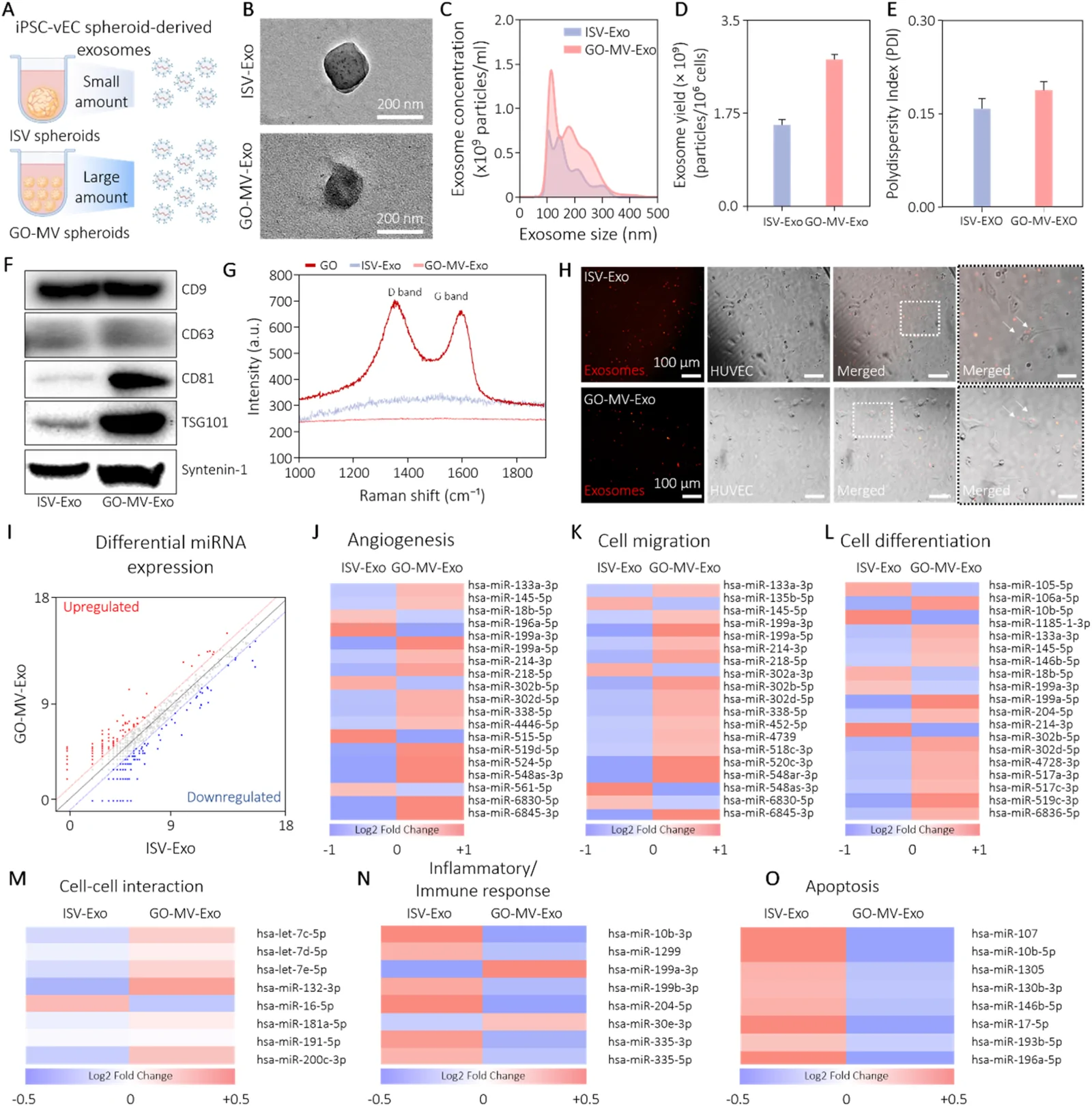
**Characterization and miRNA profiling of exosomes derived from conventional and GO-engineered iPSC-vEC spheroids. (A)** Schematic illustration of exosome isolation from conventional iPSC-vEC spheroids (ISV spheroids) and GO-engineered multiple iPSC-vEC spheroids (GO-MV spheroids). **(B)** Representative TEM images of ISV-Exo and GO-MV-Exo. Scale bars, 200 nm. **(C)** NTA-based particle-size distribution profiles of ISV-Exo and GO-MV-Exo. **(D)** Quantification of exosome yield normalized to the initially seeded cell number. **(E)** Polydispersity index (PDI) of ISV-Exo and GO-MV-Exo determined by dynamic light scattering (DLS). **(F)** Representative Western blot analysis of exosomal markers CD9, CD63, TSG101, and Syntenin-1 and the negative marker Calnexin in ISV-Exo and GO-MV-Exo, together with densitometric quantification. **(G)** Raman spectra of GO, ISV-Exo, and GO-MV-Exo showing the characteristic D and G bands of GO and the absence of prominent GO-associated Raman features in the isolated exosome preparations. **(H)** Representative fluorescence, bright-field, and merged images showing the uptake of fluorescently labeled ISV-Exo and GO-MV-Exo by HUVECs after 24 h of incubation. Dashed boxes indicate the regions shown at higher magnification, and white arrows indicate representative fluorescent exosome signals within HUVECs. Scale bars: 100 μm. **(I)** Scatter plot showing differential miRNA expression between ISV-Exo and GO-MV-Exo, with upregulated and downregulated miRNAs indicated. **(J-O)** Heatmaps showing differentially expressed miRNAs associated with (J) angiogenesis, **(K)** cell migration, **(L)** cell differentiation, **(M)** cell-cell interaction, **(N)** immune response, and **(O)** apoptosis. Data are presented as mean ± SD from three independent biological replicates (n = 3), with three technical replicates per condition.

The molecular characteristics of the isolated vesicles were subsequently evaluated by Western blotting. Both ISV-Exo and GO-MV-Exo expressed the exosomal markers CD9, CD63, TSG101, and Syntenin-1, while Calnexin was used as a negative marker for non-exosomal cellular contamination (Fig. 6F). Raman spectroscopy was further performed to assess potential GO contamination. Although the characteristic D and G bands were detected in the GO control, these prominent GO-associated features were not evident in the isolated ISV-Exo or GO-MV-Exo preparations (Fig. 6G), supporting the absence of detectable GO nanosheet contamination. To further verify the interaction of GO-engineered exosomes with recipient endothelial cells, an exosome uptake assay was performed using HUVECs. After 24 h of incubation, fluorescently labeled ISV-Exo and GO-MV-Exo were detected in association with HUVECs, as shown by the fluorescence and merged images (Fig. 6H). The magnified regions further showed fluorescent exosome signals within the cellular regions, indicating that both ISV-Exo and GO-MV-Exo were taken up by recipient HUVECs. These findings confirm the cellular internalization of the isolated exosomes and support their availability for subsequent interaction with endothelial cells.

We further examined whether GO engineering altered the molecular cargo of iPSC-vEC-derived exosomes. miRNA sequencing revealed distinct miRNA expression patterns between ISV-Exo and GO-MV-Exo, as demonstrated by the differential expression analysis (Fig. 6I). Functional categorization of the differentially expressed miRNAs identified groups associated with angiogenesis, cell migration, cell differentiation, cell-cell interaction, immune response, and apoptosis (Fig. 6J–O). Notably, several miRNAs associated with angiogenesis, including hsa-miR-133a-3p, hsa-miR-199a-3p, and hsa-miR-214-3p, were enriched in GO-MV-Exo. Similarly, miRNAs associated with endothelial cell migration, including hsa-miR-133a-3p, hsa-miR-199a-5p, and hsa-miR-302b-5p, showed increased representation. Differentially expressed miRNAs related to cell differentiation, such as hsa-miR-106a-5p, hsa-miR-199a-3p, and hsa-miR-302b-5p, were also identified (Fig. 6J–L). Additional differentially expressed miRNAs were associated with cell-cell interaction, immune response, and apoptosis (Fig. 6M–O). Collectively, GO-engineered iPSC-vEC spheroids generated increased quantities of exosomes with characteristic vesicular morphology and exosomal marker expression, while maintaining no detectable GO-associated Raman signature. The altered miRNA cargo further revealed enrichment of miRNAs associated with angiogenesis, migration, differentiation, cell-cell interaction, immune regulation, and apoptosis, supporting the potential of GO-MV-Exo as a vascular regenerative exosome platform.

### 2.7 GO-engineered iPSC-vEC-derived exosomes enhance endothelial migration and angiogenic activity

To determine whether the enhanced exosome production and altered miRNA cargo from GO-engineered iPSC-vEC spheroids translated into improved endothelial function, we next evaluated the biological activity of ISV-Exo and GO-MV-Exo using HUVECs. Analysis of angiogenesis-associated factors revealed increased levels of bFGF, EGF, VEGF, VEGFR2, TGF-β, and HGF in GO-MV-Exo compared with ISV-Exo (Fig. 7A), indicating an enhanced pro-angiogenic cargo profile. We first examined the effects of the exosomes on endothelial cell migration using a scratch wound-healing assay. Both ISV-Exo and GO-MV-Exo promoted wound closure compared with untreated control cells, with GO-MV-Exo producing the greatest increase in wound coverage at 12 and 24 h (Fig. 7B and C). These results demonstrate the enhanced pro-migratory activity of GO-MV-Exo toward endothelial cells. We next evaluated endothelial tube formation. Treatment with ISV-Exo and GO-MV-Exo enhanced the formation of interconnected endothelial networks compared with untreated control cells (Fig. 7D). Quantitative analysis showed that GO-MV-Exo significantly increased total tube length, total branching length, number of nodes, and number of loops compared with the control and ISV-Exo groups (Fig. 7E–H).

**Fig. 7:**
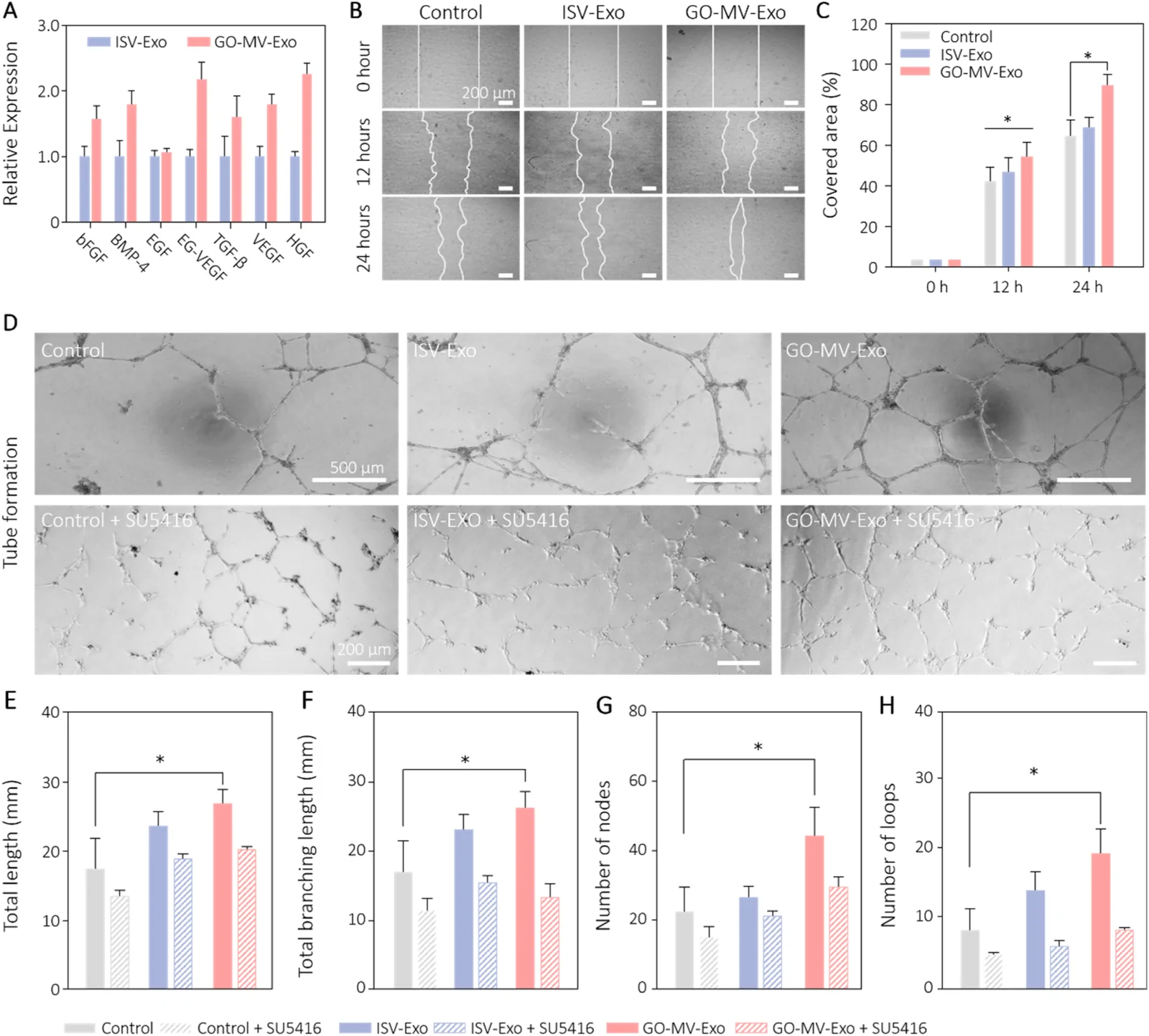
**GO-MV-Exo enhances endothelial migration and angiogenic network formation through VEGFR2-dependent signaling. (A)** Relative levels of angiogenesis-associated factors in ISV-Exo and GO-MV-Exo. **(B)** Representative images of HUVEC scratch wound-healing assays at 0, 12, and 24 h following treatment with Control, ISV-Exo, or GO-MV-Exo. Scale bars, 200 μm. **(C)** Quantification of wound coverage area at 0, 12, and 24 h. **(D)** Representative images of HUVEC tube formation following treatment with Control, ISV-Exo, or GO-MV-Exo in the presence or absence of the VEGFR2 inhibitor SU5416. Scale bars, 500 μm for the upper panels and 200 μm for the lower panels. **(E-H)** Quantification of total tube length, total branching length, number of nodes, and number of loops, respectively. Data are presented as mean ± SD from three independent biological replicates (n = 3), with three technical replicates per condition.

To further examine the contribution of VEGFR2 signaling to the GO-MV-Exo-mediated angiogenic response, HUVECs were treated with the VEGFR2 inhibitor SU5416 in combination with the respective exosome preparations. SU5416 attenuated the enhanced tube-forming activity induced by GO-MV-Exo, as reflected by reductions in the quantitative parameters of endothelial network formation (Fig. 7D–H). These findings support the involvement of VEGFR2-dependent signaling in the enhanced angiogenic activity of GO-MV-Exo. Collectively, GO-MV-Exo exhibited enhanced pro-migratory and pro-angiogenic activity compared with ISV-Exo, while VEGFR2 inhibition attenuated the GO-MV-Exo-mediated endothelial response, supporting a VEGFR2-dependent mechanism.

### 2.8 GO-engineered iPSC-vEC spheroid-derived exosomes accelerate in vivo skin regeneration

Building on the enhanced in vitro angiogenic activity of endothelial lineage exosomes, we next investigated whether iPSC-vEC spheroid-derived exosomes could promote vascularized skin regeneration in vivo. A full-thickness excisional wound model was established on the dorsal skin of rats, followed by local administration of ISV-Exo and GO-MV-Exo at days 0, 3, 7, and 10 (Fig. 8A). Wound healing progression was monitored until day 14. Representative macroscopic images revealed distinct differences in wound closure dynamics among the experimental groups (Fig. 8B). Untreated wounds exhibited delayed closure, irregular boundaries, and persistent exposure of the wound bed. ISV-Exo treatment resulted in moderate improvement, characterized by partial wound contraction and accelerated re-epithelialization. In contrast, GO-MV-Exo treatment produced markedly accelerated healing, with rapid wound contraction and progressive restoration of the skin surface. Quantitative analysis confirmed these observations (Fig. 8C and D). Both exosome-treated groups demonstrated progressive reductions in residual wound area, but GO-MV-Exo consistently produced the greatest reduction at each time point. By day 14, wounds treated with GO-MV-Exo achieved approximately 98% closure, compared with 95% in the ISV-Exo group and 94% in untreated controls. Importantly, GO-MV-Exo treatment was associated with qualitative features of improved skin regeneration, including enhanced tissue texture, reduced scab formation, and a more homogeneous skin surface.

**Fig. 8:**
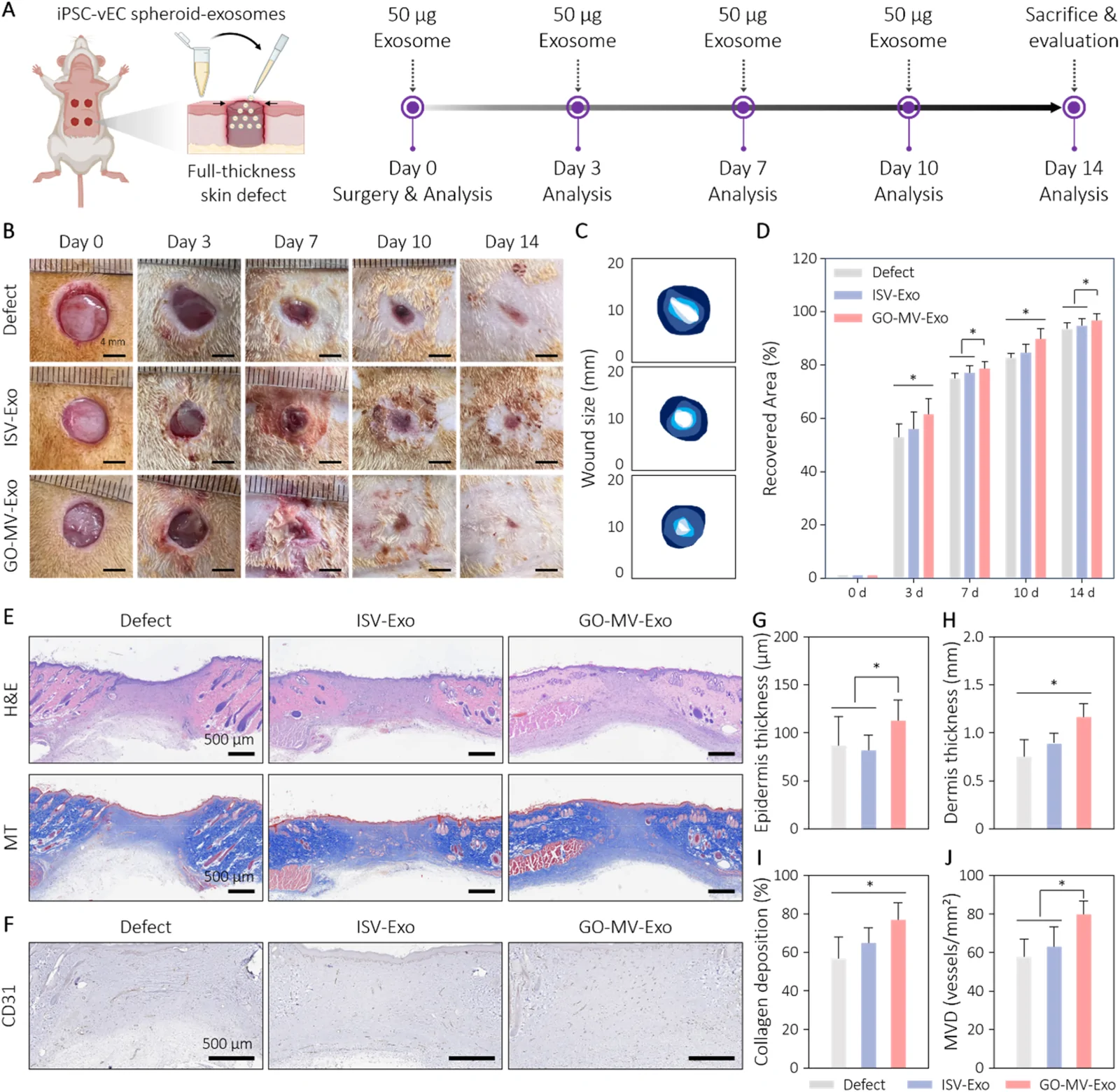
**GO-engineered iPSC-vEC spheroid-derived exosomes accelerate in vivo skin regeneration. (A)** Schematic illustration of the experimental design for evaluating the therapeutic efficacy of exosomes derived from GO-engineered iPSC-vEC spheroids in a rat full-thickness skin wound model (n = 6 animals per group). Exosomes derived from conventional iPSC-vEC spheroids (ISV-Exo) or GO-engineered multiple iPSC-vEC spheroids (GO-MV-Exo) were locally administered to dorsal wounds at days 0, 3, 7, and 10, and wound healing was monitored until day 14. **(B)** Representative images showing wound closure in untreated control, ISV-Exo-treated, and GO-MV-Exo-treated groups. **(C)** Quantitative analysis of wound closure over time based on residual wound area measurements. **(D)** Percentage wound closure demonstrating significantly accelerated healing in the GO-MV-Exo group compared with the ISV-Exo-treated and untreated control groups. **(E)** Representative hematoxylin and eosin (H&E)- and Masson's trichrome-stained sections of regenerated wound tissues at day 14. **(F)** Representative CD31 immunohistochemical staining showing CD31-positive vascular structures (brown) within the regenerated wound tissue. **(G)** Quantitative analysis of granulation tissue thickness. **(H)** Quantitative analysis of epidermal thickness. **(I)** Quantification of collagen deposition, expressed as the percentage of collagen-positive area relative to the total analyzed wound tissue area. **(J)** Quantification of CD31-positive MVD, expressed as the number of CD31-positive vessels per unit tissue area (vessels/mm^2^). Quantitative analyses were performed using n = 6 animals per group. (For interpretation of the references to colour in this figure legend, the reader is referred to the Web version of this article.)

Histological analysis further confirmed the enhanced regenerative efficacy of GO-MV-Exo at day 14. H&E-stained sections showed more advanced reconstruction of the wound tissue in the GO-MV-Exo-treated group, with a more developed granulation tissue layer and more complete restoration of the epidermal architecture compared with the untreated control and ISV-Exo groups (Fig. 8E). Masson's trichrome staining revealed more extensive blue-stained collagen-rich regions within the regenerated dermis of GO-MV-Exo-treated wounds (Fig. 8E). CD31 immunohistochemical staining further demonstrated enhanced vascular regeneration in the GO-MV-Exo-treated wounds, as evidenced by a greater abundance of brown CD31-positive vascular structures throughout the regenerated tissue (Fig. 8F). Notably, repeated local administration of exosomes did not result in apparent adverse histological features within the regenerated wound tissue at day 14. In particular, histological examination did not reveal overt evidence of inflammatory cell infiltration, tissue necrosis, or abnormal inflammatory tissue reactions in the exosome-treated groups. Instead, exosome-treated wounds showed enhanced epidermal restoration, granulation tissue formation, collagen deposition, and CD31-positive vascularization compared with untreated controls. Quantitative histomorphometric analysis supported these observations, showing the greatest granulation tissue thickness in the GO-MV-Exo group (Fig. 8G). Epidermal thickness was also increased following exosome treatment, with GO-MV-Exo exhibiting the highest value and ISV-Exo showing an intermediate response compared with the untreated control (Fig. 8H). The collagen fibers appeared more abundant throughout the wound bed, indicating enhanced extracellular matrix formation and remodeling. Consistent with the histological observations, quantitative analysis demonstrated the highest collagen deposition in the GO-MV-Exo group, while ISV-Exo showed an intermediate level compared with the untreated control (Fig. 8I). These findings suggest that GO-MV-Exo promotes more effective matrix deposition during the remodeling phase of wound healing. Quantitative analysis showed that CD31-positive microvessel density was highest in the GO-MV-Exo group, followed by the ISV-Exo and untreated control groups (Fig. 8J). Taken together, these histological and immunohistochemical findings indicate that GO-MV-Exo promotes coordinated skin regeneration by enhancing granulation tissue formation, re-epithelialization, collagen deposition, and neovascularization, supporting more effective structural and vascular restoration of full-thickness skin wounds.

The superior regenerative outcomes observed with GO-MV-Exo are consistent with their elevated pro-angiogenic signaling identified in vitro. Compared with GO-M-Exo, GO-MV-Exo exhibited higher levels of vascular growth factors and angiogenic regulators, likely promoting more efficient neovascularization and tissue remodeling during wound repair. Endothelial cells play a central role in tissue regeneration by coordinating angiogenesis with stromal remodeling and re-epithelialization, and the lineage-specific cues provided by GO-MV-Exo appear to enhance this process. Collectively, these findings demonstrate that GO-MV-Exo exhibits superior in vivo regenerative efficacy, significantly accelerating vascularized skin repair compared with ISV-Exo. These results highlight the critical contribution of endothelial lineage commitment to exosome-mediated vascularization and functional tissue regeneration in full-thickness skin defects.

## 3 Discussion

This study presents a GO-mediated multi-spheroid nanoengineering strategy that advances iPSC-derived exosomes as a scalable cell-free therapeutic platform by simultaneously enhancing exosome yield and angiogenic bioactivity [36,40]. In the broader context of regenerative medicine, this work addresses a critical shift from stem cell-based therapies toward exosome-based cell-free therapeutics, where clinical translation is often limited not only by insufficient functional potency but also by the lack of robust and scalable production platforms. Here, we show that GO-mediated modulation of early aggregation behavior reproducibly reprograms spheroid organization from a single dominant aggregate to multiple uniformly sized spheroids per well, thereby establishing a structurally optimized exosome-producing microenvironment. Importantly, this architectural reconfiguration was not merely a morphological change; it translated directly into increased exosome yield, enhanced endothelial functionality, and improved vascularized tissue regeneration. To our knowledge, this study represents a previously unexplored GO-based multi-spheroid engineering strategy for synchronously enhancing both the quantity and quality of therapeutic exosomes derived from iPSC-based spheroid systems.

A central structural finding of this study was that GO incorporation consistently induced the formation of multiple smaller spheroids rather than a single large aggregate. This architectural shift is highly relevant to both cell function and exosome biogenesis, as large spheroids frequently develop hypoxic, nutrient-deprived, and metabolically heterogeneous cores, which can compromise cell survival, differentiation fidelity, and secretory activity [[41], [42], [43]]. In contrast, GO-induced multi-spheroid organization likely improves the surface area-to-volume ratio, facilitates more efficient mass transport, and supports a more uniform biochemical and mechanical microenvironment [44]. Although the precise mechanism underlying this aggregation shift remains to be fully elucidated, it is plausible that GO nanosheets influence early multicellular assembly by modulating cell-cell and cell-material interactions during spheroid nucleation [45,46]. The high surface area and oxygen-containing functional groups of GO may alter local adhesion dynamics through protein adsorption, redistribution of interfacial forces, or transient regulation of compaction behavior, thereby favoring the formation of multiple stable aggregates rather than unrestricted coalescence into a single spheroid [47,48]. From a translational perspective, this finding is particularly important because it suggests that simple nanoengineered control over spheroid architecture can be leveraged as a scalable upstream design parameter for exosome production, rather than relying solely on downstream isolation or concentration methods.

Compared with conventional spheroid-generation methods, the GO-mediated strategy offers a distinct approach to controlling spheroid architecture through material-induced regulation of cell aggregation. Hanging-drop and ultra-low-attachment methods are simple but generally favor individual spheroid formation, whereas microwell- and microfluidic-based platforms provide greater control over spheroid size and throughput but often require specialized devices [49,50]. Dynamic suspension and bioreactor systems are more scalable, although shear stress, aggregate fusion, and size heterogeneity remain concerns [51]. In contrast, GO promotes the formation of multiple smaller spheroids within a conventional culture format by modulating early cell-cell and cell-material interactions, providing a relatively simple upstream strategy to increase exosome-producing units while maintaining structural control.

Beyond its structural effects, GO-mediated nanoengineering also exerted a pronounced influence on cellular phenotype and lineage-specific maturation. The formation of multiple smaller spheroids following GO incorporation may be associated with changes in the interfacial microenvironment during the early aggregation process. GO possesses abundant oxygen-containing functional groups and a high surface area, which can influence surface energy and protein adsorption at the cell–material interface. Such changes may alter the availability and organization of adhesion-related proteins and thereby modulate cell–cell and cell–matrix interactions during initial aggregation. Consistent with this possibility, GO-M spheroids exhibited altered expression of CDH1 and ITGB1, together with changes in F-actin organization and YAP1 expression, suggesting that GO-mediated modulation of adhesion and mechanotransduction contributes to the distinct spheroid architecture. These changes may favor the formation of multiple smaller aggregates rather than the compact single spheroid observed in conventional cultures. GO-treated spheroids exhibited improved viability under 3D conditions and enhanced expression of endothelial markers at both the protein and transcriptional levels, indicating that GO did not merely preserve cell survival but actively supported the establishment of a more functionally relevant endothelial microenvironment [52,53]. This effect may arise through several complementary mechanisms. GO is known to adsorb biomolecules and growth factors, thereby enriching the local presentation of differentiation cues at the cell interface. In parallel, its nanoscale physicochemical properties may influence integrin signaling, cytoskeletal organization, and mechanotransductive pathways that regulate lineage commitment and maturation [54,55]. Within spheroid systems, such effects are likely amplified because differentiation is governed not only by soluble factors but also by spatial organization, intercellular tension, and matrix-like microenvironmental feedback. These findings therefore support the concept that GO functions not only as a structural additive but as an active microenvironmental regulator capable of shaping both spheroid morphology and lineage-specific cell state.

Importantly, the structural and phenotypic changes induced by GO were accompanied by substantial improvements in paracrine output and exosome function. Exosome biogenesis is tightly coupled to the metabolic status, differentiation state, and microenvironmental organization of the donor cell population [[56], [57], [58]]. In this study, GO-mediated multi-spheroid formation increased the number of viable exosome-producing units, while enhanced endothelial differentiation further enriched the functional state of the donor cells. As a result, GO-engineered spheroids produced higher exosome yields when normalized to the initially seeded cell number, while maintaining nanoscale particle-size distributions and relatively low polydispersity. The presence of canonical exosomal markers, together with the absence of detectable Calnexin contamination and the lack of prominent GO-associated Raman signatures, further supported the quality of the isolated vesicle preparations. Quantitative densitometric analysis of exosomal markers provided additional confirmation of the vesicular characteristics of the isolated fractions. The uptake of exosomes by recipient cells further supported their ability to interact with and enter target cells, providing a functional basis for their subsequent biological effects. More importantly, exosomes derived from GO-engineered spheroids exhibited enriched pro-angiogenic molecular signatures, including factors such as ANG-1, ANG-2, HGF, bFGF, and VEGF, which are closely associated with endothelial activation, vascular remodeling, and angiogenic sprouting [59,60]. The miRNA profiling further revealed that GO engineering altered exosomal miRNA cargo associated with angiogenesis, cell migration, cell differentiation, cell–cell interaction, immune regulation, and apoptosis, while KEGG enrichment highlighted pathways including focal adhesion, regulation of the actin cytoskeleton, VEGF, MAPK, Wnt, TGF-β, and mTOR signaling. These findings reinforce a key conceptual point of this study: exosome quality is strongly determined by the structural and biological context of the donor spheroid microenvironment, and nanoengineering this context can simultaneously improve both yield and therapeutic potency. Consistent with this concept, Huang et al. combined HGF-overexpressing MSCs with a herringbone microfluidic culture platform to simultaneously increase exosome production and therapeutic cargo enrichment, resulting in enhanced angiogenesis and wound healing [61]. Recent evidence further supports that modulation of the three-dimensional donor-cell microenvironment can simultaneously improve exosome yield and functional potency [62].

The enhanced molecular signatures of GO-derived exosomes translated into robust functional angiogenic responses in both in vitro and in vivo settings. Importantly, pharmacological inhibition of VEGFR2 using SU5416 attenuated the enhanced tube-forming activity induced by GO-engineered exosomes, providing functional evidence for the involvement of VEGFR2-dependent signaling in the angiogenic response. Together, these data strengthen the mechanistic link between the altered molecular cargo of GO-engineered exosomes and their enhanced angiogenic activity, rather than attributing the observed endothelial responses solely to increased exosome abundance. Exosomes derived from GO-engineered spheroids significantly improved endothelial migration and capillary-like network formation, two hallmark processes required for effective neovascularization [63]. These results suggest that GO-engineered exosomes actively modulate endothelial cytoskeletal dynamics, intercellular communication, and ECM-associated remodeling pathways that support angiogenic progression [64,65]. Importantly, these in vitro findings were functionally validated in full-thickness skin wound models, where exosomes derived from GO-engineered iPSC spheroids and iPSC-vEC spheroids accelerated wound closure and enhanced vascularized tissue repair. Beyond exosome-based approaches, recent biomaterial- and nanocomposite-based strategies have shown strong wound-healing potential, and their combination with therapeutic exosomes may further enhance regenerative outcomes through synergistic effects on vascular regeneration, re-epithelialization, extracellular matrix remodeling, and antibacterial protection [66,67]. Similarly, a multifunctional living hydrogel incorporating blood-derived components and black phosphorus quantum dots promoted wound repair by coordinating hemostasis, oxygen delivery, antibacterial activity, and tissue regeneration [68]. Moreover, MSC-derived exosomes incorporated into an antibacterial double-network hydrogel exhibited prolonged local retention and enhanced angiogenesis and tissue regeneration, demonstrating the potential of biomaterial-assisted exosome delivery [69].

Since successful skin regeneration depends heavily on timely angiogenesis to restore oxygen delivery, nutrient exchange, stromal activation, and epithelial remodeling, the observed improvement in wound healing is mechanistically consistent with the enhanced vascular signaling identified in our in vitro assays [70,71]. Thus, GO-mediated exosome engineering not only improves vesicle production but also generates functionally superior secretomes capable of driving clinically relevant tissue repair responses.

A particularly important finding of this study was that the GO-based strategy remained effective when extended from undifferentiated iPSC spheroids to iPSC-derived vascular endothelial cell (vEC) spheroids, further strengthening both the mechanistic and translational significance of the platform. The increased exosome production and enhanced endothelial activity observed following GO engineering of iPSC-vEC spheroids further demonstrate that this strategy is not restricted to undifferentiated iPSC spheroids but can be retained following lineage-specific differentiation. While exosomes derived from undifferentiated iPSC spheroids already exhibited pro-angiogenic activity, exosomes from GO-engineered endothelial lineage spheroids showed further enhancement of vascular-specific signaling and regenerative efficacy. Consistent with this functional distinction, the distinct miRNA profiles observed between GO-M-Exo and GO-MV-Exo indicate that lineage commitment may further shape the molecular cargo of GO-engineered exosomes. This comparison provides important mechanistic insight by demonstrating that lineage commitment and microenvironmental nanoengineering act synergistically to define exosome bioactivity. In other words, the therapeutic performance of exosomes is not determined solely by the donor cell source, but also by how that donor population is structurally organized and developmentally programmed. This is an important conceptual advance because it suggests that exosome manufacturing platforms should be designed not only to maximize vesicle number, but also to strategically tune cell state and lineage specificity in order to generate functionally optimized therapeutic products.

Despite the favorable short-term cytocompatibility observed at GO concentrations up to 20 μg/mL in this study, the long-term biosafety of GO-based nanoengineering remains an important consideration for clinical translation. GO-associated biological responses are known to depend on physicochemical parameters such as lateral sheet size, oxidation degree, surface chemistry, colloidal dispersion, aggregation state, dose, exposure route, and protein corona formation [72]. In particular, residual or aggregated GO nanosheets may interact with immune cells, persist within local tissues, accumulate in reticuloendothelial organs, or undergo size-dependent renal and immune-mediated clearance [73]. Therefore, short-term cytocompatibility data alone are not sufficient to fully exclude potential long-term toxicity, delayed inflammatory responses, tissue accumulation, or incomplete clearance.

In the present platform, GO was used as an upstream spheroid-engineering cue to regulate spheroid assembly and exosome production, rather than as a therapeutic cargo intended for direct administration. The final therapeutic material administered in vivo was the isolated exosome fraction, which limits direct host exposure to GO. To minimize residual GO carryover during exosome preparation, the collected conditioned medium was centrifuged at 2000 × g for 30 min at 4 °C as a primary clarification step to remove cells, residual GO aggregates, and debris, followed by filtration through a 0.45 μm syringe filter to further reduce residual GO aggregates and large particulate contaminants. In addition, Raman spectroscopic analysis and TEM observation of the isolated exosome preparations showed no detectable GO-specific Raman signatures or GO-like sheet structures, supporting the absence of detectable residual GO in the final isolated exosome fraction.

Nevertheless, because precipitation-based extracellular vesicle isolation methods can co-isolate non-vesicular particles, protein aggregates, soluble protein complexes, or material-associated contaminants, the possibility of trace residual GO below the detection limits of Raman and TEM analyses cannot be completely excluded [74]. Such trace GO-associated materials could potentially affect downstream therapeutic applications by influencing recipient-cell responses, inflammatory activation, angiogenic bioactivity, batch-to-batch consistency, and safety profiles after repeated administration. Therefore, future translational development should incorporate orthogonal purification and release-testing strategies, including size-exclusion chromatography, density-gradient separation, tangential-flow filtration, Raman spectroscopy, TEM/EDS, AFM, UV-Vis absorbance analysis, elemental/carbon analysis, and labeled GO tracing. Long-term in vivo studies evaluating biodistribution, clearance kinetics, repeated-dose toxicity, immunotoxicity, organ accumulation, and local inflammatory responses will be essential to establish clinically acceptable safety and quality-control criteria for scalable GO-engineered exosome manufacturing.

Although this study demonstrates that GO-mediated multi-spheroid formation enhances exosome yield and angiogenic bioactivity, several practical challenges must be addressed for clinical-scale translation. Large-scale spheroid production will require precise control of spheroid size and number, GO dispersion, cell seeding density, oxygen/nutrient transport, and culture geometry. During scale-up into high-throughput plates, stirred suspension systems, or bioreactor-based formats, additional variables such as shear stress, spheroid fusion, heterogeneous GO distribution, and batch-dependent spheroid maturation may affect product consistency [12,20]. Exosome purification also remains a major translational bottleneck. Although exosomes in this study were isolated using pre-clearing centrifugation, filtration, and a precipitation-based reagent, clinical manufacturing will require more scalable and selective GMP-compatible purification workflows, such as tangential-flow filtration, size-exclusion chromatography, density-gradient separation, or integrated combinations of these approaches [13,16]. In parallel, standardized release criteria should be established, including particle concentration, size distribution, exosomal marker expression, residual impurity levels, sterility, endotoxin content, and functional potency [75]. Finally, batch-to-batch reproducibility will be essential for the clinical application of GO-engineered exosomes. Variability may arise from iPSC passage number, differentiation efficiency, GO lot-to-lot physicochemical properties, spheroid heterogeneity, culture duration, and exosome isolation efficiency. Therefore, future translational studies should define critical process parameters and critical quality attributes linking spheroid architecture, exosome yield, molecular cargo, and angiogenic potency. Establishing these manufacturing and quality-control frameworks will be necessary to translate the GO-mediated platform into a reproducible, scalable, and clinically applicable exosome production system [75].

A further limitation of this study is that the local retention, biodistribution, and systemic safety of repeatedly administered exosomes were not directly evaluated. Although repeated local exosome administration promoted wound closure, tissue reconstruction, collagen deposition, and CD31-positive neovascularization without apparent adverse histological findings in the regenerated wound tissue, these findings primarily support local regenerative efficacy rather than definitive systemic safety. Therefore, future studies should include fluorescent exosome tracking and serum inflammatory cytokine analysis to evaluate biodistribution, clearance kinetics, local retention, systemic responses, and overall safety after repeated administration. Taken together, despite these remaining translational safety considerations, this study establishes GO-based multi-spheroid nanoengineering as a promising strategy for generating high-yield, high-potency angiogenic exosomes from iPSC-derived spheroid systems and supports upstream microenvironmental engineering as a powerful design principle for next-generation exosome therapeutics in vascularized tissue regeneration, ischemic repair, and chronic wound healing.

## 4 Conclusion

In conclusion, this study establishes GO-mediated multi-spheroid nanoengineering as an effective and scalable upstream strategy for the production of high-yield, functionally potent angiogenic exosomes. By modulating early cellular aggregation, GO reproducibly reorganized spheroid architecture into multiple uniform units, thereby increasing exosome yield and enhancing pro-angiogenic bioactivity without compromising vesicle integrity or cytocompatibility. This approach was applicable to both undifferentiated iPSC spheroids and endothelial-committed iPSC-vEC spheroids, while lineage-directed differentiation further strengthened vascular-specific signaling and wound-healing efficacy. These findings indicate that exosome quality is determined not only by the donor-cell source, but also by the structural organization and developmental state of the donor population. Nevertheless, the precise mechanisms underlying GO-mediated spheroid reorganization and the long-term biosafety of GO require further investigation. Future studies incorporating scalable GMP-compatible purification processes, standardized manufacturing criteria, and long-term in vivo safety and efficacy assessments will be essential to translate this platform into a reproducible and clinically applicable cell-free therapeutic strategy for vascularized tissue regeneration and wound healing.

## 5 Experimental section

### 5.1 GO preparation

GO nanosheets (∼0.5 um 1% aqueous dispersion, GRAPHENEALL, #HGO6-S05, Republic of Korea) were dispersed in sterile deionized water to prepare stock solutions. Suspensions were sonicated for uniform dispersion and autoclaved before use. Working concentrations (0, 5, 10, and 20 μg/mL) were freshly prepared in culture medium immediately prior to spheroid generation.

### 5.2 GO characterization

The physicochemical properties of graphene oxide (GO) nanosheets were characterized using transmission electron microscopy (TEM), zeta potential analysis, Fourier-transform infrared (FT-IR) spectroscopy, X-ray photoelectron spectroscopy (XPS), Raman spectroscopy, and atomic force microscopy (AFM). Prior to characterization, GO suspensions were diluted in deionized water and briefly sonicated to obtain a homogeneous dispersion and minimize aggregation. The morphology and lateral size of GO nanosheets were observed by TEM (JEM-ARM200F NEO ARM, JEOL Ltd., Japan). For TEM sample preparation, a drop of the diluted GO suspension was deposited onto a carbon-coated copper grid, allowed to adsorb for several minutes, and dried at room temperature before imaging. The surface charge of GO was evaluated by zeta potential analysis using zeta potential analyzer (ELSZneo, Otsuka Electronics, Japan). GO suspensions were diluted to an appropriate concentration in deionized water and sonicated immediately before measurement. Measurements were performed at room temperature to assess the dispersion stability of GO in aqueous suspension. FT-IR spectroscopy (Spectrum 3, PerkinElmer Inc., USA) was conducted to examine the chemical functional groups of GO, including oxygen-containing groups such as hydroxyl, carbonyl, carboxyl, and epoxy groups. XPS (K-ALPHA+, Thermo Fisher Scientific, USA) was performed to analyze the surface elemental composition and chemical bonding states of GO. Survey spectra and high-resolution C 1s and O 1s spectra were acquired to evaluate carbon- and oxygen-related chemical species. Raman spectroscopy (NRS-5100, JASCO, Japan) was used to investigate the structural characteristics of GO. Raman spectra were collected from dried GO samples to identify the characteristic D and G bands, and the intensity ratio of the D and G bands was calculated as an indicator of defect density and structural disorder. AFM (NX20, Park Systems, Republic of Korea) was conducted to further examine the surface morphology and nanosheet thickness of GO. For AFM analysis, diluted GO suspensions were drop-cast onto mica or silicon substrates and dried under ambient conditions. Nanosheet thickness and surface topography were analyzed from AFM height profiles.

### 5.3 iPSC culture

Human iPSCs (hFSiPS3-1, passages 24-35, Korea National Institute of Health, Republic of Korea) were cultured in mTeSR1 medium (Stemcell Technologies, #85850, Canada) supplemented with 10 µM ROCK inhibitor (Y-27632 dihydrochloride, Tocris Bioscience, #1254, UK) on 1% Matrigel-coated plates (Corning, hESC-qualified, #354277, USA) in DMEM/F12 (Gibco, #11330-032, USA). iPSCs were maintained and cultured in a humidified cell incubator at 37 ¡ÆC with 5% CO©ü. Cultures were maintained according to established protocols in 6-well plates. At 70% confluency, cells were passaged every 4 days at a 1:10 ratio using Accutase with 0.5 mM EDTA (Stemcell Technologies, #07920, Canada).

### 5.4 GO-mediated iPSCspheroid formation

Human iPSCs were enzymatically dissociated into single cells and seeded at a density of 1 × 10^4^ cells per well in ultra-low attachment U-bottom plates in mTeSR1 medium supplemented with 10 μM Y-27632 dihydrochloride. For GO-mediated spheroid formation, the sterilized GO solution was dispersed in culture medium at the desired concentration and sonicated immediately before use. The GO-containing medium was added to dissociated iPSCs at the time of aggregation. Control spheroids were generated under identical conditions without GO supplementation. Cells were cultured under static conditions to allow spontaneous 3D assembly. Spheroids were cultured in a humidified incubator at 37 °C with 5% CO_2_. Spheroid formation and morphology were monitored for 5 days, after which conditioned medium was collected for exosome isolation and functional analyses.

### 5.5 GO-mediated iPSC-vEC spheroid formation

Human iPSCs were dissociated and seeded at a density of 1 × 10^4^ cells per well in mTeSR1 medium supplemented with 10 μM Y-27632 dihydrochloride to enhance cell survival. Cells were allowed to aggregate for 24 h under static conditions to initiate spheroid formation. Spheroids were cultured in a humidified incubator at 37 °C with 5% CO_2_. Human iPSCs were subsequently differentiated into vECs using a stepwise differentiation protocol consisting of mesoderm priming, endothelial induction, and maturation phases. On Day 0, lateral mesoderm induction was initiated using N2B27-based priming medium. The basal medium consisted of a 1:1 mixture of DMEM/F12 (49.5 mL) supplemented with GlutaMAX (500 μL) and Neurobasal medium (49 mL) supplemented with N2 (500 μL) and B27 (500 μL). These two media were combined in equal proportions to generate the final N2B27 formulation. Mesoderm induction was achieved by supplementing the medium with CHIR99021 (6 μM; 0.6 μL/mL), BMP4 (25 ng/mL; 0.5 μL/mL), and 10 μM ROCK inhibitor (1 μL/mL). No media change was performed during the initial 3-day mesoderm priming phase to allow stable lineage commitment. On Day 3, endothelial cell induction was initiated using StemPro-34 serum-free medium (SFM), with daily medium replacement. The induction medium consisted of StemPro-34 supplemented with VEGFA (200 ng/mL; 10 μL/mL), forskolin (2 μM; 0.2 μL/mL), basic fibroblast growth factor (bFGF, 20 ng/mL; 0.4 μL/mL), and SB431542 (10 μM; 1 μL/mL). This stage promoted endothelial lineage specification and vascular gene activation. From Day 6 onward, cells entered the maturation and expansion phase. The maturation medium consisted of StemPro-34 supplemented with VEGFA (50 ng/mL; 2.5 μL/mL), bFGF (20 ng/mL; 0.4 μL/mL), and ascorbic acid (50 μg/mL; 5 μL/mL). On Day 6 only, 1% Matrigel was added to support ECM remodeling and endothelial stabilization. The maturation medium was refreshed regularly to maintain optimal growth conditions. Differentiation was continued until Day 20, at which point mature vascular endothelial spheroids were collected. For GO-mediated iPSC-vEC spheroid formation, the sterilized GO solution was dispersed in culture medium at the desired concentration and sonicated to ensure uniform suspension prior to cell seeding. The GO-containing medium was added to dissociated iPSCs at the time of aggregation and differentiation was initiated. Conditioned medium was harvested after 20 days of differentiation for downstream exosome isolation and functional analyses.

### 5.6 Cell viability and cytotoxicity analysis

To assess spheroid viability and cytotoxicity, live/dead staining was performed using Calcein AM and Ethidium Homodimer-1 (Live/Dead Viability/Cytotoxicity Kit, Thermo Fisher Scientific, #L3224, USA) according to the manufacturer's instructions. Spheroids were incubated with 2 μM Calcein AM and 4 μM Ethidium Homodimer-1 in PBS at 37 °C for 30 min. Following staining, spheroids were washed with PBS and imaged using a confocal fluorescence microscope. For three-dimensional visualization of the spheroids, confocal z-stack images were acquired by collecting sequential optical sections throughout the entire spheroid volume. Viable cells exhibited green fluorescence, whereas dead cells exhibited red fluorescence. The acquired z-stack images were reconstructed and analyzed using ImageJ to evaluate the distribution and proportion of viable and dead cells within the spheroids. Quantitative analysis of spheroid viability and cytotoxicity was performed using more than 10 spheroids per group from at least three biological replicates.

Cell viability of iPSCs and iPSC spheroids was further evaluated using a WST-1 assay according to the manufacturer's instructions. For monolayer cultures, iPSCs were seeded in 96-well plates and treated with GO at concentrations of 0, 5, 10, and 20 μg/mL. For 3D spheroid cultures, iPSCs were seeded in ultra-low-attachment plates and treated with the same GO concentrations. Cell viability was assessed on days 1 and 3 after seeding. Absorbance was measured at 450 nm using a microplate reader, and values were normalized to the control group. Quantitative analysis was performed using at least three biological replicates.

### 5.7 Immunofluorescence staining

Whole-mount spheroids were fixed in 4% paraformaldehyde for 20 min, followed by three washes with 1X phosphate-buffered saline (PBS) for 5 min each. Fixed spheroids were permeabilized with 0.1% Triton X-100 in PBS for 30 min, followed by three additional PBS washes. Samples were then blocked with 1% bovine serum albumin (BSA; Cell Signaling Technologies, #9998S, USA) for 1 h. Subsequently, spheroids were incubated with BSA-diluted primary antibodies for 24 h at 4 °C. Primary antibodies used were anti-CD31 (Invitrogen, #MA5-15336, USA) and anti-vWF (Novus Biologicals, #NB600-586-0.1 mL, USA). After washing three times with 1X PBS, samples were stained with PBS-diluted secondary antibodies, including Cy3 AffiniPure Goat Anti-Mouse IgG (H + L) (Jackson ImmunoResearch, #115-165-003, USA) and Fluorescein (FITC) AffiniPure Goat Anti-Rabbit IgG (H + L) (Jackson ImmunoResearch, #111-095-003, USA). Following three additional PBS washes, cell nuclei were counterstained with 4,6-diamidino-2-phenylindole (DAPI, Thermo Fisher Scientific, #D1306, USA). Spheroids were mounted using ProLong Gold Antifade Reagent (Invitrogen, #P36935, USA). Fluorescence images were acquired using a laser confocal scanning microscope, and relative fluorescence intensity was quantified using ImageJ software.

### 5.8 Quantitative real-time PCR

Total RNA was extracted using TRIzol Reagent (Invitrogen, # 15596026, USA) according to the manufacturer's instructions. RNA concentration and purity were determined using a NanoDrop Lite spectrophotometer (Thermo Scientific, USA). Complementary DNA (cDNA) was synthesized from 2 μg of total RNA using the GoScript Reverse Transcription System (Promega, #A501D, USA) following the manufacturer's protocol. Quantitative real-time PCR (qRT-PCR) was performed using GoTaq qPCR Master Mix (2X) (Promega, #A6001, USA) on a Bio-Rad real-time PCR system. Gene-specific forward and reverse primers were custom-designed and synthesized by Macrogen (Republic of Korea). Primer sequences for vWF, TEK, EGR, and the housekeeping gene GAPDH are listed in Table 1. Amplification reactions were performed according to the manufacturer's recommended cycling conditions. Melt curve analysis was conducted to confirm amplification specificity. Relative gene expression levels were normalized to GAPDH and calculated using the comparative 2^−ΔΔCT^ method. Results are presented as fold changes relative to the control group.

**Table 1:** List of genes and primer sequences used for qRT-PCR.

Genes	Forward sequence	Reverse sequence
GAPDH	5′-AACGGGAAGCCCATCACC-3′	5′- CAGCCTTGGCAGCACCAG-3′
vWF	5′-CCTTGAATCCCAGTGACCCTGA-3′	5′-GGTTCCGAGATGTCCTCCACAT-3′
TEK	5′-GGTCAAGCAACCCAGCCTTTTC-3′	5′-CAGGTCATTCCAGCAGAGCCAA-3′
EGR	5′-AGCAGCACCTTCAACCCTCAGG-3′	5′-GAGTGGTTTGGCTGGGGTAACT-3′
CDH1	5′-TGCCTGAGAACGAGGCTAAC-3′	5′-GGTGTATACAGCCTCCCACG-3′
ITGB1	5′-GCCGCGCGGAAAAGATGAA-3′	5′-TGAGCAAACACACAGCAAACT-3′
HIF1A	5′-GTCTGAGGGGACAGGAGGAT-3′	5′-CTCCTCAGGTGGCTTGTCAG-3′
NOS3	5′-GACCCACTGGTGTCCTCTTG-3′	5′-CCCGAACACACAGAACCTGA-3′
RAB27A	5′-TACCCGCCAACGACCCAAAA-3′	5′-CTTTTTGTCTGGCTCGCCC-3′
RAB27B	5′-ATAAGTAGCTGTCCCCGTGC-3′	5′-TTTCCTTCCCCTTCCCAAGC-3′
ALIX	5′-ACTCTCCCCAAGGAGGTGTT-3′	5′-TGGTACTCAGCATTGGCCTG-3′
SDCBP	5′-GCTCTCTCAATACATGGGGCT-3′	5′-GCTACCAACTGCCCCTGAAG-3′
YAP1	5′-GCCATGACTCAGGATGGAGA-3′	5′-GGTTCATGGCAAAACGAGGG-3′
BAX	5′-TTCATCCAGGATCGAGCAGG-3′	5′-CACTCGCTCAGCTTCTTGGT-3′
BCL2	5′-GATGGGATCGTTGCCTTATGC-3′	5′-ACTTCCTCTGTGATGTTGTATTTTT-3′
TSG101	5′-CTGACCCTCTGCCTGCG-3′	5′-ACACCATTTTCTTGAGCTGGC-3′
CASP3	5′-GCGGATGGGTGCTATTGTGA-3′	5′-ACACAGCCACAGGTATGAGC

### 5.9 Nitric oxide detection

Intracellular nitric oxide (NO) levels in day-20 conventional iPSC-vEC spheroids (ISV) and GO-engineered iPSC-vEC spheroids (GO-MV) were evaluated using DAF-FM™ Diacetate (4-Amino-5-Methylamino-2′,7′-difluorofluorescein diacetate) (Invitrogen, #D23844, USA). Spheroids were incubated with 5 μM DAF-FM Diacetate for 60 min to allow intracellular loading of the fluorescent probe. Following incubation, spheroids were washed with PBS to remove excess probe and further incubated in fresh buffer/medium to allow complete de-esterification of the intracellular diacetate. Fluorescence images were then acquired using a fluorescence/confocal microscope. The fluorescence intensity of DAF-FM was quantified from the acquired images and used as a relative measure of intracellular NO levels.

### 5.10 Exosome isolation from iPSC and iPSC-vEC spheroid-derived conditioned medium

Conditioned medium was collected from day 5 spheroids generated from undifferentiated iPSCs and day 20 iPSC-vEC spheroids. Prior to collection, spheroids were cultured in serum-free medium for 48 h to minimize contamination from exogenous extracellular vesicles. The collected conditioned medium was first centrifuged at 2000 × g for 30 min at 4 °C to remove cells and cellular debris. The supernatant was then passed through a 0.45 μm syringe filter to further remove residual cellular debris, large aggregates, and particulate contaminants, including potential residual GO aggregates. The filtered conditioned medium was subsequently subjected to exosome isolation using the Total Exosome Isolation Reagent (Thermo Fisher Scientific, #4478359, USA) according to the manufacturer's instructions. Briefly, the isolation reagent was added to the clarified and filtered conditioned medium and incubated at 4 °C overnight to precipitate extracellular vesicles. Samples were then centrifuged at 10,000 × g for 2 h at 4 °C. The resulting exosome pellet was resuspended in sterile 1X PBS and stored at −80 °C until further characterization and functional analyses.

### 5.11 Exosome characterization

#### 5.11.1 TEM

IS-Exo, ISV-Exo, GO-M-Exo, GO-MV-Exo exosome suspensions were placed on carbon-coated copper grids and allowed to adsorb for 10 min. Excess liquid was removed, and samples were negatively stained with 2% uranyl acetate. Grids were air-dried and imaged using a transmission electron microscope at an appropriate accelerating voltage. Representative images were captured to assess vesicle morphology and size.

#### 5.11.2 Nanoparticle tracking analysis (NTA)

Exosome samples were diluted in sterile PBS to achieve optimal particle concentration. Particle size and concentration were analyzed using NTA according to the manufacturer's instructions, and average values were calculated using the instrument software.

#### 5.11.3 Dynamic light scattering and PDI analysis

The hydrodynamic particle size and polydispersity index (PDI) of the isolated exosomes were determined by dynamic light scattering using a Zeta-PSA Surface Zeta Potential & Particle Size Analyzer. IS-Exo, GO-M-Exo, ISV-Exo, and GO-MV-Exo samples were submitted to the facility, and measurements were performed by the facility operator according to the manufacturer's standard procedure.

#### 5.11.4 Raman spectroscopy

Raman spectroscopy was performed to characterize graphene oxide (GO) and to evaluate the potential presence of GO-associated signals in the isolated exosome preparations. GO, IS-Exo, ISV-Exo, GO-M-Exo, and GO-MV-Exo samples were analyzed by Raman spectroscopy. The Raman spectra were recorded over the characteristic Raman shift range, and the D and G bands of GO were used as reference signals for comparison with the exosome preparations.

#### 5.11.5 Exosome uptake assay in HUVECs

Exosome uptake by HUVECs was evaluated using fluorescently labeled exosomes. ISV-Exo and GO-MV-Exo were labeled with the ExoGlow™-Membrane EV Labeling Kit (System Biosciences, #EXOGP100A-1, USA) according to the manufacturer's instructions. Following removal of excess fluorescent dye, the labeled exosomes were added to HUVECs and incubated for up to 48 h. At the indicated time points, cells were gently washed with PBS to remove unbound exosomes and subsequently fixed for fluorescence imaging. Exosome uptake was evaluated based on the intracellular fluorescence signal observed in HUVECs.

#### 5.11.6 Exosome yield normalization

Exosome production was quantified by nanoparticle tracking analysis (NTA) and normalized to the number of cells initially used for spheroid generation. The total number of exosome particles was calculated from the NTA-derived particle concentration and the final exosome suspension volume. Exosome yield was subsequently expressed as the number of particles per 10^6^ initially seeded cells (particles/10^6^ cells) to enable comparison of exosome production efficiency between the different spheroid groups.

#### 5.11.7 miRNA sequencing and bioinformatic analysis

Exosomal miRNA profiles were analyzed to identify differences in miRNA cargo between conventional and GO-engineered spheroid-derived exosomes. Total RNA was isolated from the exosome preparations using Total Exosome RNA & Protein Isolation Kit (Invitrogen, #4478545, USA) and small RNA sequencing was performed. Differentially expressed miRNAs between the experimental groups were identified based on the sequencing data and visualized using heatmap and differential-expression analyses. Differentially expressed miRNAs were subsequently categorized according to their reported biological functions, including angiogenesis, cell migration, cell differentiation, cell–cell interaction, immune response, and apoptosis. For functional enrichment analysis, experimentally supported target genes of the differentially expressed miRNAs were identified using miRNet 2.0 with *Homo sapiens* as the species and miRTarBase v9.0 as the target database. KEGG pathway enrichment was performed using the hypergeometric test. Adjusted p-values were used to account for multiple testing, and enriched pathways were visualized using bubble plots. Bubble size represents the number of target genes, while pathway enrichment was plotted as −log_10_(adjusted p-value).

#### 5.11.8 Exosome lysis and protein quantification

Exosome suspensions were diluted in 1X PBS and lysed by adding an equal volume of RIPA buffer (Thermo Scientific, #89901, USA) supplemented with protease inhibitor cocktail (cOmplete, Mini Protease Inhibitor Cocktail, Roche, #11836153001, USA). Samples were incubated on ice at 4 °C for 15 min, followed by centrifugation at 12,000 × *g* for 30 min at 4 °C. The supernatant containing total exosomal proteins was collected for downstream analysis. Protein concentrations were measured using a bicinchoninic acid (BCA) protein assay (Pierce BCA Protein Assay Kit, Thermo Scientific, #23225, USA) following the manufacturer's instructions. Protein concentrations were calculated using a standard curve generated from serially diluted BSA.

#### 5.11.9 Western blot analysis

Equal amounts of exosomal protein (30 μg per lane) were mixed with SDS loading buffer, denatured at 95 °C for 10 min, and separated on 10% SDS-PAGE gels. Proteins were transferred to nitrocellulose membranes using a wet transfer system. Subsequently, membranes were stained with Ponceau S to confirm uniform protein transfer, followed by blocking with 5% skim milk in 1x TBST for 1 h at room temperature (20-25 °C). Membranes were incubated overnight at 4 °C with primary antibodies against CD9 (Abcam, #ab236630, USA), CD63 (Abcam, #ab134045, USA), CD81 (Abcam, #ab79559, USA), TSG101 (Abcam, #ab125011, USA), Calnexin (Abcam, #ab133615, USA), and β-Actin (Cell Signaling Technology, #4970S, USA), Syntenin-1 (Cell Signaling Technology, # 32485, USA). After washing with 1x TBST, membranes were incubated with horseradish peroxidase (HRP)-conjugated secondary antibodies (Anti-rabbit IgG, HRP-linked Antibody, Cell Signaling Technology, #7074, USA) for 2 h at room temperature. Protein bands were detected using chemiluminescence reagents (Immobilon Western Chemiluminescent HRP Substrate, Millipore, #P90719, USA) and visualized with a chemiluminescence imaging system (Bio-Rad, ChemiDoc MP Imaging System, USA). CD9 and TSG101 were used as positive exosomal markers, Calnexin served as a negative control to assess cellular contamination, and β-Actin was used where appropriate as a loading control.

#### 5.11.10 Growth factor array

The angiogenesis-related protein cargo of exosomes was profiled using a membrane-based human angiogenesis antibody array (Human Angiogenesis Array Q1; Ray Biotech, Peachtree Corners, GA, USA) according to the manufacturer's instructions. Briefly, exosome samples were prepared at equivalent protein input (50 μg) and, where necessary, lysed using the lysis buffer provided in the kit to enable detection of intra-vesicular and membrane-associated proteins. Array membranes were initially blocked with the supplied blocking buffer to minimize non-specific binding and then incubated with exosome lysates under gentle agitation. After thorough washing with the kit-provided wash buffers, membranes were incubated sequentially with the provided biotin-conjugated detection antibody cocktail and HRP-conjugated streptavidin. Chemiluminescent signals were developed using the kit's substrate reagents and captured using a digital chemiluminescence imaging system.

### 5.12 Migration assay

Human umbilical vein endothelial cells (HUVECs) were cultured in Endothelial Growth Medium (EGM; Lonza) at 37 °C in a humidified cell incubator with 5% CO_2_. Cells were seeded into 12-well plates and grown to approximately 90-100% confluence. A linear cell-free scratch area was created in the cell monolayer using a sterile 1000 μL pipette tip, and detached cells were gently removed by washing twice with PBS. Fresh EGM containing the respective treatments was added immediately. The experimental groups were control (EGM only), IS-Exo, GO-M-Exo, ISV-Exo, and GO-MV-Exo. Exosomes were added to each well immediately after scratch creation at a final concentration of 50 μg/mL exosomal protein, as determined by the BCA assay, and the same exosomal protein concentration was used for all exosome-treated groups. Prior to treatment, purified exosome preparations were centrifuged to pellet the exosomes. The PBS-containing supernatant was completely removed, and the resulting exosome pellets were resuspended in fresh Endothelial Growth Medium (EGM) to the desired concentration and immediately applied to the cells. Images of the wound area were captured at 0, 12, and 24 h using an inverted phase-contrast microscope. Wound closure was quantified by measuring the remaining wound area at each time point using image analysis software, and the percentage of wound closure was calculated.

### 5.13 Angiogenesis assay

The in vitro angiogenic potential of exosomes was evaluated using a Matrigel-based tube formation assay. Briefly, growth factor-reduced Matrigel (Corning, #354230, USA) was coated onto 48-well plates and allowed to polymerize at 37 °C for 30 min in a humidified atmosphere containing 5% CO_2_. HUVECs cultured in Endothelial Growth Medium were harvested and seeded onto Matrigel-coated wells at a density of 4 × 10^4^ cells per well in 150 μL of culture medium containing 50 μg of exosomal protein, as quantified using a BCA protein assay, under the following treatment conditions: control (EGM only), IS-Exo, ISV-Exo, GO-M-Exo, and GO-MV-Exo derived from both iPSC spheroids and iPSC-vEC spheroids. Prior to treatment, purified exosome preparations were centrifuged to pellet the exosomes. The PBS-containing supernatant was completely removed, and the resulting exosome pellets were resuspended in fresh Endothelial Growth Medium (EGM) to the desired concentration and immediately applied to the cells. Cells were incubated under standard culture conditions (37 °C, 5% CO_2_) for 12 h to allow capillary-like network formation. Representative microscopic images were captured using an inverted phase-contrast microscope. Quantitative analysis of tube formation was performed using image analysis software, and angiogenic parameters, including total tube length, total branching length, number of nodes, and number of loops, were measured. All experiments were performed in triplicate, and data were expressed as mean ± standard deviation.

### 5.14 Pharmacological inhibition of VEGFR2 signaling

For VEGFR2 inhibition experiments, a Matrigel-based tube formation assay was performed. Briefly, HUVECs were seeded onto polymerized Matrigel-coated 24-well plates at a density of 3 × 10^4^ cells per well in 150 μL of Endothelial Growth Medium (EGM). Cells were treated with IS-Exo, GO-M-Exo, ISV-Exo, or GO-MV-Exo at 50 μg of exosomal protein per well. For VEGFR2 inhibition, SU5416 (5 μM) was added concurrently with the respective exosome treatments. Control groups received EGM without exosomes, with or without SU5416. Cells were incubated at 37 °C in a humidified atmosphere containing 5% CO_2_ for 20 h to allow capillary-like network formation. Representative microscopic images were then acquired, and angiogenic network formation was quantitatively evaluated based on total tube length, total branching length, number of nodes, and number of loops.

### 5.15 In vivo animal wound healing analysis

The animal study was approved by the Institutional Animal Care and Use Committee (IACUC) of Chonnam National University (CNU IACUC-YB-2025-110). Male Sprague-Dawley (SD) rats (200-300 g, 7-8 weeks old) were purchased from Damul Science, Korea. The animals were housed under standard laboratory conditions at 22 ± 2 °C and 50 ± 10% relative humidity, with a 12 h light/dark cycle and free access to food and water. The therapeutic efficacy of exosomes derived from both iPSC spheroids and iPSC-vEC spheroids was evaluated using a full-thickness excisional wound model in rats. All animal experiments were conducted in accordance with institutional guidelines for animal care and use. Briefly, healthy 7-8-week-old rats were fully anesthetized by intramuscular injection of ketamine (Yuhan Corporation, Seoul, Korea) and xylazine hydrochloride (Rompun; Bayer HealthCare, Korea), and their dorsal skin was shaved and disinfected. A full-thickness circular skin defect, 8 mm in diameter, was created using a sterile biopsy punch, extending through the epidermis and dermis. The animals were randomly divided into five groups: PBS-treated control, IS-Exo, GO-M-Exo, ISV-Exo, and GO-MV-Exo. Each experimental group included six rats (n = 6 per group), which served as biological replicates for the in vivo wound-healing analysis. Exosome treatments, 50 μg of exosomal protein per wound, were administered locally immediately after surgery on day 0 and repeated on days 3, 7, and 10. The control group received an equal volume of PBS. At the experimental endpoint, the animals were euthanized by overdose of Zoletil and Rompun in accordance with institutional animal care and use guidelines, and wound tissues were harvested for further analysis. Digital photographs of the wound area were taken at days 0, 3, 7, 10, and 14 using a standardized imaging setup with a scale reference. Wound closure was quantified using ImageJ software, and the percentage of recovered area was calculated as: Recovered Area (%) = [(Initial wound area - Current wound area)/Initial wound area] × 100.

### 5.16 Histological and immunohistochemical analyses

At day 14, wound tissues were harvested (n = 6 per group), fixed in 10% neutral buffered formalin, embedded in paraffin, and sectioned for histological analysis. Hematoxylin and eosin (H&E) staining was performed to evaluate overall tissue regeneration, including epidermal thickness and granulation tissue thickness. Masson's trichrome staining was used to assess collagen deposition within the regenerated dermal tissue. Histomorphometric measurements were performed using ImageJ software. Epidermal thickness was measured from the epidermal surface to the dermal-epidermal junction, while granulation tissue thickness was measured within the regenerated wound bed. Collagen deposition was quantified as the percentage of blue-stained collagen-positive area relative to the total analyzed wound tissue area. Neovascularization was evaluated by immunohistochemical staining for CD31. Following deparaffinization, rehydration, and antigen retrieval, tissue sections were incubated with a rabbit monoclonal anti-CD31 antibody (EPR17259, Abcam, #ab182981, USA), followed by Goat Anti-Rabbit IgG H&L (HRP) secondary antibody (Abcam, #ab6721, USA). CD31-positive endothelial structures were visualized using DAB and counterstained with hematoxylin. Distinct CD31-positive vascular structures within the regenerated dermis and granulation tissue were counted as individual microvessels. MVD was calculated as the number of CD31-positive vessels divided by the analyzed tissue area and expressed as vessels/mm^2^. Multiple representative regions were analyzed for each animal, and the mean value was used for statistical analysis.

### 5.17 Statistical analysis

All data are presented as mean ± standard deviation (SD) from at least three independent biological replicates. Comparisons between two groups were performed using a two-tailed unpaired Student's *t*-test, and comparisons among three or more groups were performed using one-way ANOVA followed by Tukey's post hoc test. Significance levels are indicated as: *p < 0.05, **p < 0.01, ***p < 0.001, and ****p < 0.0001.

## CRediT authorship contribution statement

**Woochan Kim:** Writing – review & editing, Writing – original draft, Visualization, Software, Resources, Methodology, Investigation, Data curation, Conceptualization. **Harshita Sharma:** Writing – review & editing, Writing – original draft, Methodology, Investigation, Data curation. **Dream Kim:** Writing – review & editing, Investigation. **Shinyull Lee:** Writing – review & editing, Investigation. **Sefali Bhakuni:** Writing – review & editing, Investigation. **Jangho Kim:** Writing – review & editing, Visualization, Validation, Supervision, Resources, Project administration, Investigation, Funding acquisition, Formal analysis, Conceptualization.

## Funding

This work was supported by a 10.13039/501100003725National Research Foundation of Korea (NRF) grant funded by the Korean government (10.13039/501100014188MSIT) (RS-2024-00337706, RS-2025-02216206, RS-2020-NR049568, and RS-2022-NR067329). This work was also supported by the 10.13039/501100014189Korea Institute of Planning and Evaluation for Technology in Food, Agriculture and Forestry (10.13039/501100014189IPET) through the Agriculture and Food 10.13039/100021494Convergence Technologies Program for Research Manpower Development, funded by the 10.13039/501100003624Ministry of Agriculture, Food and Rural Affairs (10.13039/501100003624MAFRA) (RS-2024-00397026). This work was supported by the 10.13039/501100014189Korea Institute of Planning and Evaluation for Technology in Food, Agriculture and Forestry (10.13039/501100014189IPET) through the Companion Animal Intractable Disease Overcoming R&D Project, funded by the 10.13039/501100003624Ministry of Agriculture, Food and Rural Affairs (10.13039/501100003624MAFRA) (RS-2026-25519399). This research was supported by the Commercialization Promotion Agency for R&D Outcomes (10.13039/501100023443COMPA) funded by the 10.13039/501100014188Ministry of Science and ICT (10.13039/501100014188MSIT) (RS-2025-25430866).

## Declaration of competing interest

The authors declare that they have no known competing financial interests or personal relationships that could have appeared to influence the work reported in this paper.

## Data Availability

Data will be made available on request.
