# Supplementary material for: Graphene oxide-induced multiple iPSC spheroid formation for enhanced production of angiogenic exosomes accelerating tissue regeneration
Source: Mater Today Bio. 2026 Oct 5;41:103723. doi: 10.1016/j.mtbio.2026.103723 (PMC13682188; doi:10.1016/j.mtbio.2026.103723)
Supplement: Supplementary file 1 [file mmc1.docx]

**Graphene Oxide-Induced Multiple iPSC Spheroid Formation for Enhanced Production of Angiogenic Exosomes Accelerating Tissue Regeneration**

Woochan Kim^a,b,c,+^, Harshita Sharma^a,b,c,+^, Dream Kim^a,b,c^, Shinyull Lee ^a,b,c^, Sefali Bhakuni^a,b,c^, Jangho Kim ^a,b,c,d,*^

^a^ Department of Convergence Biosystems Engineering, Chonnam National University, Gwangju, 61186, Republic of Korea

^b^ Department of Rural and Biosystems Engineering, Chonnam National University, Gwangju, 61186, Republic of Korea

^c^ Interdisciplinary Program in IT-Bio Convergence System, Chonnam National University, Gwangju 61186, Republic of Korea

^d^ Institute of Nano-Stem Cells Therapeutics, NANOBIOSYSTEM Co., Ltd; Gwangju 61003, Republic of Korea

* Corresponding author: Jangho Kim ([rain2000@jnu.ac.kr](mailto:rain2000@jnu.ac.kr))

+ Woochan Kim and Harshita Sharma contributed equally to this work.

**Figure S1. Physicochemical characterization of GO nanosheets. (A)** Representative TEM images showing the sheet-like morphology and lateral dimensions of GO nanosheets. **(B)** Zeta potential of GO nanosheets dispersed in aqueous solution, indicating the surface charge characteristics of GO. **(C)** FT-IR spectrum showing characteristic oxygen-containing functional groups of GO. **(D)** XPS survey spectrum confirming the elemental composition of GO. **(E,F)** High-resolution C 1s and O 1s XPS spectra showing the deconvoluted chemical bonding states of carbon and oxygen species. **(G)** Raman spectrum displaying the characteristic D and G bands of GO, indicating its defect-rich graphitic structure. **(H)** AFM image and **(I)** corresponding height profile showing the surface morphology and nanoscale thickness of GO.

**Figure S2. Optimization of GO concentration and effects on iPSC spheroid characteristics. (A)** Representative images of iPSC spheroid formation following treatment with 0, 5, 10, and 20 µg/mL GO. Scale bar, 2 mm. **(B, C)** Quantification of iPSC viability under monolayer culture at days 1 and 3, respectively, following treatment with different concentrations of GO. **(D, E)** Quantification of iPSC spheroid viability at days 1 and 3, respectively, following treatment with different concentrations of GO. **(F)** Quantification of spheroid area of conventional iPSC spheroids and GO-M spheroids at days 1, 3, and 5. **(G)** Quantification of spheroid circularity index at days 1, 3, and 5. **(H)** Quantification of cell viability of conventional iPSC spheroids and GO-M spheroids at days 1, 3, and 5. **(I)** Relative expression of exosome biogenesis- and secretion-associated markers (ALIX, SDCBP, TSG101, RAB27A, and RAB27B) in conventional iPSC spheroids and GO-M spheroids. **(J)** Relative expression of apoptosis-associated markers (CASP3, BCL2, and BAX) in conventional iPSC spheroids and GO-M spheroids.
